# Site-Selective Electrochemical Oxidation of Glycosides

**DOI:** 10.1021/acscatal.2c06318

**Published:** 2023-01-31

**Authors:** Marios Kidonakis, Augustin Villotet, Martin D. Witte, Sebastian B. Beil, Adriaan J. Minnaard

**Affiliations:** Stratingh Institute for Chemistry, University of Groningen, Nijenborgh 7, 9747 AG Groningen, The Netherlands

**Keywords:** glycosides, site-selective, electrosynthesis, oxidation, metal-free conditions

## Abstract

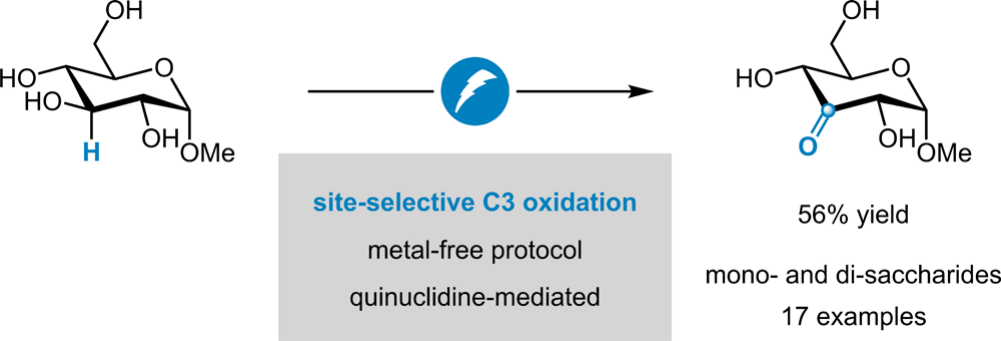

Quinuclidine-mediated electrochemical oxidation of glycopyranosides provides C3-ketosaccharides with high selectivity and good yields. The method is a versatile alternative to Pd-catalyzed or photochemical oxidation and is complementary to the 2,2,6,6-tetramethylpiperidine 1-oxyl (TEMPO)-mediated C6-selective oxidation. Contrary to the electrochemical oxidation of methylene and methine groups, the reaction proceeds without oxygen.

Carbohydrates are an important class of compounds, both in biology, in food and feed, and as raw materials for industry. The monosaccharide building blocks mostly occur in their glycopyranoside form.^1,2^ Despite the application of carbohydrates, their selective functionalization is elusive since differentiation between the virtually identical secondary hydroxyl groups is required. In synthesis, protection-group strategies are mostly applied but hamper large-scale industrial applications.

Over the past years, significant progress has been made in the site-selective modification of unprotected and partially protected carbohydrates.^3−5^ Regioselective oxidation is particularly noteworthy due to the versatile derivatization of respective ketones or carboxylic acids.^6−8^ TEMPO-mediated oxidation of glycopyranosides leads to selective oxidation of the primary C6 hydroxy group, most often producing the corresponding uronic acids.^9−11^ Palladium-catalyzed oxidation^12^ shows a strong preference for the secondary C3 hydroxy group (Figure 1). The preference for the C3 over the C2 and C4 position (Figure 1A) was rationalized based on thermodynamic and kinetic arguments.^13^

**Figure 1:**
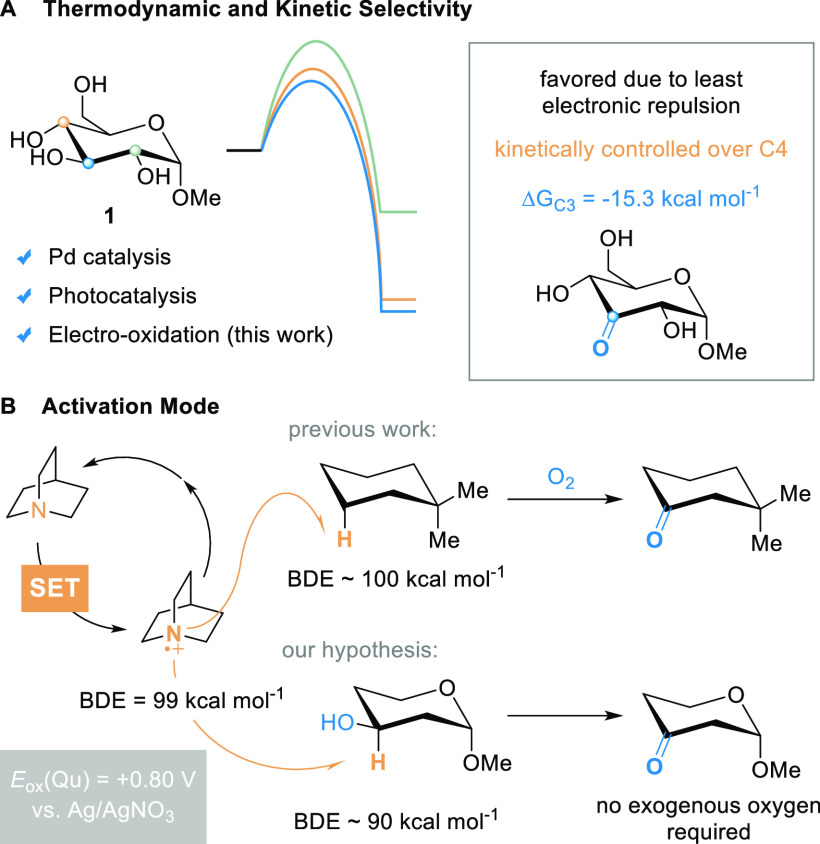
Site-selective C3 oxidation of methyl-α-D-glucopyranoside (**1**) (A). Quinuclidine-mediated activation of C–H bonds (B), inspired by Baran and co-workers.^31^

This palladium-catalyzed oxidation reaction has shown to be versatile^14−17^ and scalable;^6−8^ however, in applications, a metal-free protocol would be advantageous.

The photochemical oxidation mediated by quinuclidine and a photosensitizer fulfills this demand,^18,19^ and we showed the related selective photochemical alkylation reaction to be scalable in a continuous flow system.^20^ Work from our group and of the groups of Wendlandt, Taylor, and Wang showed that quinuclidinyl radical cations abstract preferentially the hydrogen atom at C3 of gluco-configured saccharides.^18,19,21−23^ Co-catalysts, such as borinic acids, and alternative hydrogen atom-transfer reagents can be used to alter the site selectivity.^24−26^

The electrochemical oxidation of the primary and the anomeric hydroxy group in sugars is well-described. TEMPO and 4-acetamido-TEMPO are the mediators of choice,^27,28^ and Pt- and Au-based electrodes have mainly been applied.^29^ Similar results were obtained under heterogeneous conditions, in which a TEMPO-immobilized Nafion perfluorinated film was deposited onto graphite electrodes.^30^

When looking for a suitable metal-free electrochemically driven protocol as an alternative to the photochemical procedures featuring the distinct C3 selectivity, we wondered whether the weak C3–H bond [bond dissociation energy (BDE) ∼90 kcal mol^–1^] could be selectively activated. In 2017, Baran and co-workers reported on the electrochemical quinuclidine-mediated C–H activation of nonactivated methylene and methine groups (Figure 1B).^31^ We realized that the quinuclidine radical cation can be generated at low potentials by electrochemical oxidation (*E*_ox_ = +0.80 V vs Ag/AgNO_3_)^32^ and can abstract the C3–H due to its high difference in BDE (99 kcal mol^–1^ for Qu^+^^·^),^33^ as described before.^18,21,31^ In both the electro-oxidation of methylene/methine units according to Baran et al.,^31^ and in the photochemical alcohol oxidation according to Taylor and co-workers,^18^ oxygen or superoxide traps the formed carbon-centered radical. The formed hydroperoxide (radical) reacts then subsequently to the carbonyl function.

In our electrochemical, quinuclidine-mediated oxidation of glycosides, we used methyl-α-D-glucopyranoside (**1**) as a model substrate. A substoichiometric amount of quinuclidine (Qu, 0.3 equiv), tetramethylammonium tetrafluoroborate (Me_4_NBF_4_, 1 equiv), and hexafluoroisopropanol (HFIP, 10 equiv) were dissolved in acetonitrile and a constant current was set at 5 mA. Upon electrolysis, the initial suspension of **1** turned homogeneous over 24 h. The use of graphite electrodes (*C*_gr_), being inexpensive, pleasingly led to full oxidation of **1**. After full conversion by TLC, we obtained the 3-keto product **1a** in 56% isolated yield (see Table 1, Entry 1), although the mass balance could not be confirmed unambiguously. In particular, the apparent full selectivity for the C3 position to the keto sugar demonstrated the potential of this method. Other mediators, which are known to be potent in HAT reactions, did not perform at the same level as observed for quinuclidine (see the Supporting Information, Table S1).

**Table 1:**
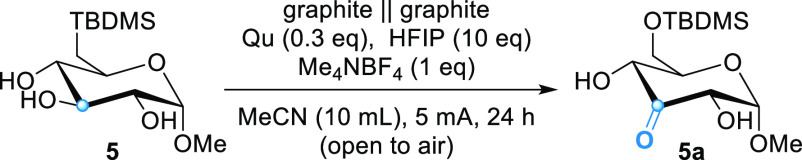
Control Experiments of the Regioselective Electrochemical Oxidation of Glucopyranoside **5**

entry	deviation from above	conv.^a^
1	none	99% (56%)^b^
2	no Qu	decomp.
3	no HFIP	decomp.
4	under argon	99% (72%)^c^
5	under oxygen	99% (76%)^c^

a Conversion determined by ^1^H NMR with 1,3,5-trimethoxybenzene as the internal standard.

b Isolated yield in parentheses.

c ^1^H NMR yield with 1,3,5-trimethoxybenzene as the internal standard.

In our proposed mechanism, the reaction starts with the formation of the quinuclidinium radical cation (Qu^+^^·^) at the anode, followed by hydrogen abstraction (HAT) of the C3–H bond (Figure 2A), following expected reactivity known for the quinuclidine/HFIP system.^31,32^ Additionally, HFIP is known to support solvation by distinct domain formation.^34^ The α-hydroxy radical **I** is a stable intermediate which forms in conjunction with protonated quinuclidine (Qu–H). Radical **I** can subsequently be trapped either by Qu^+^^·^ (green) or HFIP radical (blue), instead of oxygen or superoxide, as described in the seminal work, leading to the unstable hemiaminal cation **II** or ketal **III**. Both will quickly collapse to keto sugar **1a**, regenerating Qu–H or HFIP. Alternatively, intermediate **I** can be easily deprotonated by relative excess of the base and subsequently undergo another oxidation event close to the anode (see the Supporting Information, Figure S3). The resulting intermediate **IV** thus quickly forms the ketone product by radical recombination. The strong dependence on free quinuclidine radical cations can also be seen in the slow kinetics of the reaction, which only works at low current densities of around 1.5 mA cm^–1^. Either the quinuclidinium cation and/or HFIP can release a proton which combines at the cathode-liberating hydrogen and thus closing the overall redox reaction.

**Figure 2:**
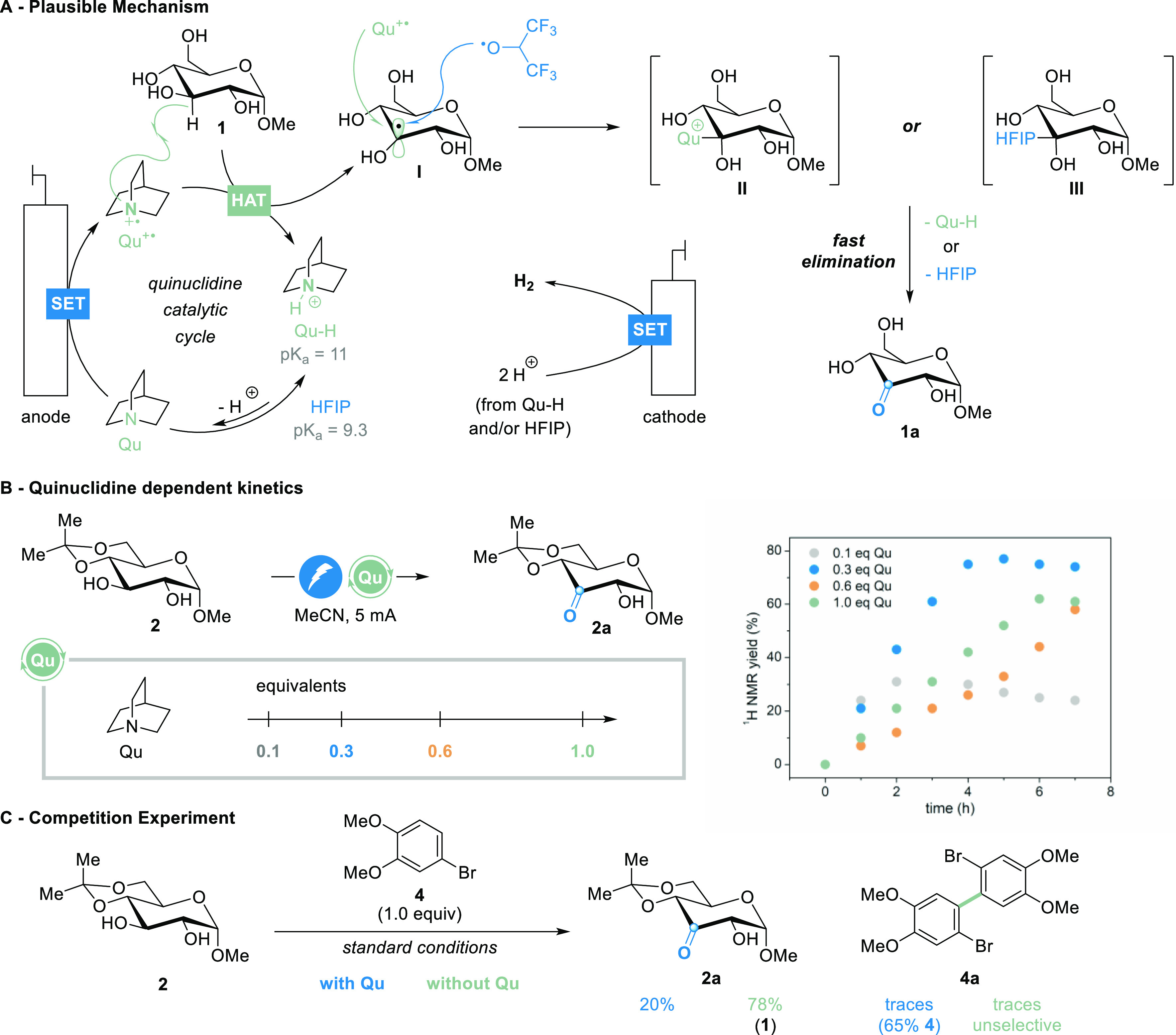
Proposed mechanism for the electrochemically mediated C3 oxidation of glycosides (A). Kinetic profiles of the quinuclidine-mediated C3-selective oxidation of glycoside **2** (B, see the Supporting Information for details). Competition experiments with electron-rich arenes, which are known to undergo oxidative coupling reactions (C).

When we were following the kinetics of the reaction by ^1^H NMR, we were surprised to see that lower or higher amounts of quinuclidine as the mediator (0.1, 0.6, or 1.0 equiv) led to lower conversions of glycoside **2** (gray, orange, and green profile, Figures 2B, and S4 in the Supporting Information) to the respective keto sugar. With an optimum of 0.3 equiv of quinuclidine (blue profile), full conversion was observed after 9 h, featuring 81% isolated yield of keto glycoside **2a**. Even more surprising to us was the catalytic amount of mediator (0.1 equiv, gray profile), which gave full conversion accompanied with pronounced product decomposition.

In the absence of an HAT mediator, graphite electrodes are prone to arene oxidation, thus resulting in dehydrodimerization reactions.^35,36^ We first tested 4-methyl veratrole (**3**) as the substrate, but the benzylic position was oxidized preferentially to the glycoside and we obtained methyl vanillin (**3′**) in 23% yield (see Figure S5). Without quinuclidine, 57% yield of the dehydrodimer (**3a**) was obtained. 4-Bromo veratrole (**4**), which has no benzylic position, undergoes oxidative dimerization at a lower rate and 17% dimer **4a** is obtained in the absence of glycoside, with 68% recovery of the substrate **4** under standard conditions (see Figure S6). When we used **4** in a competition experiment to investigate the preferential oxidation of **2**, we obtained **2a** in 20% under standard conditions together with 65% recovery of **4**. Without quinuclidine, the pH of the reaction mixture dropped significantly, and we isolated the deprotected starting material (**1**) in 78% yield, without any C3 oxidation products present. Additionally, no substrate **4** was recovered and coupling toward **4a** was unselective and occurred only in traces.

During our investigations of the reaction mixture by cyclic voltammetry, we found no clear effect of the scan rate varying between 50–200 mV s^–1^. Quinuclidine oxidation occurs between 1.24 and 1.31 V vs Ag/AgCl with regard to platinum or glassy carbon counter electrodes, respectively (see the Supporting Information, Figure S7). The quinuclidine back reduction was not clearly visible, and the oxygen reduction reaction was suppressed in the presence of HFIP.

The electrode combination developed by the Baran group,^31^ namely, reticulated vitreous carbon (RVC, a glassy carbon foam) as the anode and nickel foam as the cathode material gave full conversion, but we preferred more robust electrodes. Since initial substrate adsorption on the RVC anode was reported being important,^37^ we were pleased to find graphite being operationally simpler and similarly high-performing (Table 1, Entry 1).^38^ Importantly, mixtures with DMSO in acetonitrile (1:1) were amenable to yield the desired product due to improved solubility, which is advantageous for highly polar substrates like **1** (63% isolated yield). Protic solvents like methanol were deleterious for the reaction outcome when converting methyl glucose and no conversion was observed, despite the improved solubility. As expected, no productive reaction occurred in the absence of quinuclidine or HFIP (Table 1, Entry 2–3), supporting our mechanistic proposal. Albeit without quinuclidine, decomposition led to traces of **5** and **5a**. To probe the independence from molecular oxygen and likewise the mechanistic differentiation to the superoxide formation, we subjected **5** to electrochemical oxidation under an argon atmosphere and rigorous exclusion of oxygen. The inert conditions hardly effected the reaction and full conversion, and 72% yield was observed by ^1^H NMR analysis (Table 1, Entry 4). As a control, and to exclude the inadvertent presence of oxygen, we reproduced the C–H activation reaction reported by Baran et al. using the benchmark substrate sclareolide.^31^ As expected, this reaction clearly required oxygen, and the yield dropped dramatically to less than 5% upon exclusion of oxygen under argon (see the Supporting Information, Figure S8). A pure oxygen atmosphere had a neglectable effect on our reaction outcome (Table 1, Entry 5). We therefore conclude that the reaction progresses without any oxygen-derived species involved. A minimum of two electrons is required for the overall reaction, based on the observation of 70% conversion after 2.5 F/mol (under argon), whereas only 29% conversion was obtained after 1 F/mol.

With these results in hand, we investigated the substrate scope of the oxidation reaction, employing simple graphite electrodes. For comparison, we depict the yields for the other reported conditions utilizing the Pd-catalytic system,^12,39,40^ photochemical oxidation,^18^ as well as photochemical oxidation with manganese additives.^19^ In general, for the first time, a redox-mediated process was operational without the addition of phosphate-derived bases, which rendered a remarkable necessity in the photochemical procedures.

To facilitate solubility and chromatographic purification, partly protected glycosides were used, leading to the corresponding products in moderate to good yields (Figure 3).

**Figure 3:**
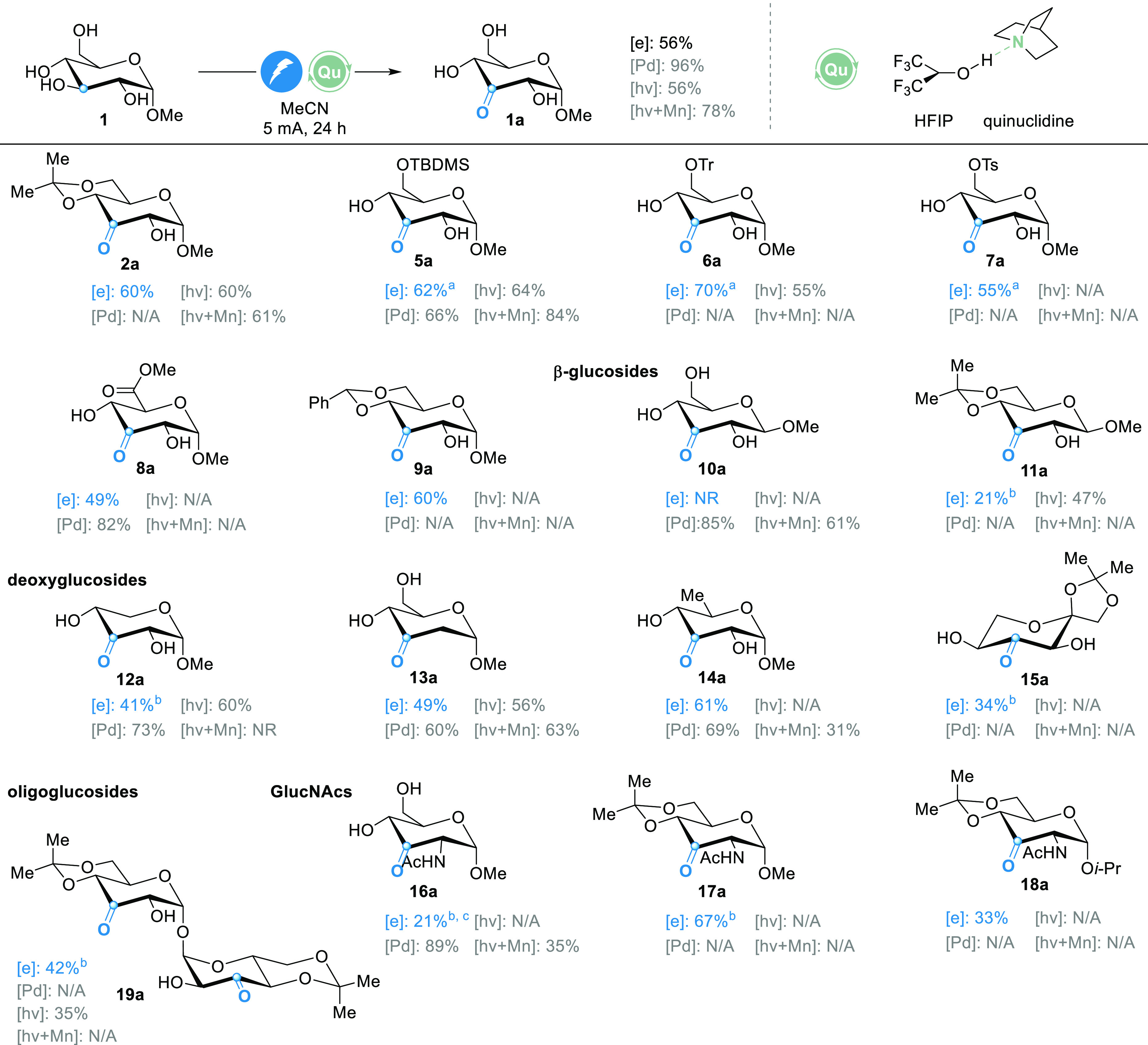
Scope of the electrochemical oxidation of mono- and disaccharides. Reaction conditions: glycoside (0.32 mmol, 1 equiv), quinuclidine (30 mol %), Me_4_NBF_4_ (1 equiv), HFIP (10 equiv), 5 mL of ACN, graphite electrodes, 5 mA (constant current, 2.00 mA/cm^2^), 16 h, yields after column chromatography. ^a^ Reaction on 0.56 mmol scale. ^b^ Reaction on 0.16 mmol scale for 24 h. ^c^ NMR yield using 1,4-difluorobenzene as the internal standard. [Pd] from ref (12, 34, 35), [hv] from ref (15), [hv + Mn] from ref (16). NR, no reaction; N/A, yield not given.

Silyl-equipped **5** was smoothly converted to ketone **5a** in 62% isolated yield. Noteworthy, the presence of a trityl group, potentially susceptible to Birch reduction,^41^ had no effect on the reaction outcome, and **6** underwent selective oxidation in 70% yield. For lipophilic substrates like **6**, column purification can be replaced by a simple aqueous work-up to receive the pure product. In addition, substrate **7**, with a tosyl group at C6, provided **7a** in 55% yield. The methyl ester of glucuronic acid **8** was smoothly oxidized in a moderate yield. 4,6-Protected monosaccharide **2** was oxidized in merely 95% ^1^H NMR yield (60% isolated yield). Surprisingly, the presence of a benzylidene function had no detrimental effect on the reaction outcome, and glucose derivative **9** provided the C3 oxidation product in 60% isolated yield. We previously observed significant acetal cleavage in the photochemical alkylation reaction,^20^ which is not operational in the electrochemical procedure. As expected, the oxidation of unprotected methyl-β-D-glucopyranoside **10** led to a complex mixture of products, but to our delight, acetal-protected β-glycoside **11** gave the desired product in an isolated yield of 21%. Hydrogen atom transfer at the anomeric position is most probably a competing reaction in β-glycosides.^42^ Deoxyglycosides xyloside **12**, 2-deoxyglycoside **13**, 6-deoxy-glycoside **14**, and acetal-protected sorbose **15** provided the respective products in moderate to good yields ranging from 34 to 61%. Oxidation of methyl *N*-acetyl glucosamine **16** was incomplete and suffered from low solubility. Acetyl-protected derivatives **17** and **18** were synthesized and led to a pleasingly high 67% yield in the oxidation reaction. Commonly, the bulkier isopropyl group in **18** increases the solubility and thus yield. However, the low 33% yield for **18a** could be explained by the steric congestion of the HAT by the quinuclidine radical cation. The method is not restricted to monosaccharides, as trehalose derivative **19** produced the twofold-oxidized product **19a** in a rewarding 42% yield. Collectively, the scope of the presented methodology resembles previous C3-selective oxidation reactions of monosaccharides. Additionally, the established methodology opens up new vistas for the selective oxidation of furanosides and other oligosaccharides, which are currently under investigation.

To illustrate the scalability of our methodology, **5** was oxidized on a 1 g scale. Product **5a** was isolated in 50% yield (Scheme 1). Initial results indicate that in the absence of supporting electrolyte, similar conversions can be obtained. The buffered system of HFIP and the (sub-)stoichiometric amounts of quinuclidine as the organic base offer potential for electrolyte-free reactions.^43,44^

**Scheme 1:**
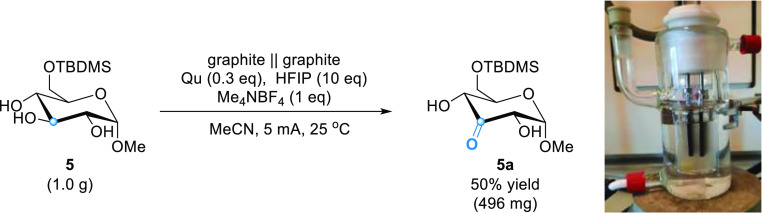
Oxidation of **5** on a Gram Scale

In conclusion, glycopyranosides can be electro-oxidized selectively at the C3 position in a quinuclidine-mediated process, which is highly complementary to the known selective electrochemical oxidation at the C6 mediated by TEMPO. Whereas the quinuclidinium radical cation attacks the weakest C–H bond in a HAT process, TEMPO in its oxoammonium form oxidizes the primary hydroxy group because it is sterically most accessible.

The use of graphite electrodes and the absence of any metal-based catalysts and sensitizers provide an asset in the scale up of this method required for its application in carbohydrate chemistry. An additional advantage is the observation that no oxygen is required for oxidation. This is a distinct advantage for electrochemistry in flow as the supply of sufficient oxygen in a flow process is technically challenging because of its low solubility and because of safety. Due to the low current density, the reactions often take very long on a large scale and future research will focus on the continuous flow electrochemical synthesis of mostly unprotected glycosides.

## Supporting Information Available

Experimental procedures; spectral analysis; and supporting discussion to experimental results (Figures S1–S8, Table S1) (PDF)

## References

[ref1] Guo Z., Chemical Synthesis of Complex Carbohydrates, in Carbohydrate Chemistry, Biology and Medical Applications. Garg H. G.; Cowman M. K.; Hales C. A.; Elsevier Science: 2008.

[ref2] Rudiger H.; Gabius H.-J., Chapter 1: The Biochemical Basis and Coding Capacity of the Sugar Code, in The Sugar Code: Fundamentals of Glycosciences. Gabius H.-J.; John Wiley & Sons: 2011.

[ref3] Jäger M.; Minnaard A. J. Regioselective modification of unprotected glycosides. Chem. Commun. 2016, 52, 656–664. 10.1039/C5CC08199H. 26568447

[ref4] Dimakos V.; Taylor M. S. Site-Selective Functionalization of Hydroxyl Groups in Carbohydrate Derivatives. Chem. Rev. 2018, 118, 11457–11517. 10.1021/acs.chemrev.8b00442. 30507165

[ref5] Witte M. D.; Minnaard A. J. Site-Selective Modification of (Oligo)Saccharides. ACS Catal. 2022, 12, 12195–12205. 10.1021/acscatal.2c03876. 36249871 PMC9552177

[ref6] Marinus N.; Tahiri N.; Duca M.; Mouthaan L. M. C. M.; Bianca S.; van den Noort M.; Poolman B.; Witte M. D.; Minnaard A. J. Stereoselective Protection-Free Modification of 3-Keto-saccharides. Org. Lett. 2020, 22, 5622–5626. 10.1021/acs.orglett.0c01986. 32635733 PMC7372562

[ref7] Zhang J.; Reintjens N. R. M.; Dhineshkumar J.; Witte M. D.; Minnaard A. J. Site-Selective Dehydroxy-Chlorination of Secondary Alcohols in Unprotected Glycosides. Org. Lett. 2022, 24, 5339–5344. 10.1021/acs.orglett.2c01992. 35848103 PMC9490796

[ref8] Marinus N.; Walvoort M. T. C.; Witte M. D.; Minnaard A. J.; Dijk H. M. V., Regioselective Palladium Catalyzed Oxidation at C-3 of Methyl Glucoside, in Carbohydrate Chemistry: Proven Synthetic Methods. Kosma P.; Wrodnigg T.; Stütz A.; CRC Press: 2021; 5.

[ref9] Bragd P. L.; Besemer A. C.; van Bekkum H. Bromide-free TEMPO-mediated oxidation of primary alcohol groups in starch and methyl α-d-glucopyranoside. Carbohydr. Res. 2000, 328, 355–363. 10.1016/S0008-6215(00)00109-9. 11072842

[ref10] van den Bos L. J.; Codée J. D. C.; Litjens R. E. J. N.; Dinkelaar J.; Overkleeft H. S.; van der Marel G. A. Uronic Acids in Oligosaccharide Synthesis. Eur. J. Org. Chem. 2007, 2007, 3963–3976. 10.1002/ejoc.200700101.

[ref11] Tiwari V.; Badavath V. N.; Singh A. K.; Kandasamy J. A highly efficient TEMPO mediated oxidation of sugar primary alcohols into uronic acids using 1-chloro-1,2-benziodoxol-3(1H)-one at room temperature. Tetrahedron Lett. 2018, 59, 2511–2514. 10.1016/j.tetlet.2018.05.021.

[ref12] Jäger M.; Hartmann M.; de Vries J. G.; Minnaard A. J. Catalytic Regioselective Oxidation of Glycosides. Angew. Chem., Int. Ed. 2013, 52, 7809–7812. 10.1002/anie.201301662. 23780519

[ref13] Wan I. C. (. S.).; Hamlin T. A.; Eisink N. N. H. M.; Marinus N.; de Boer C.; Vis C. A.; Codée J. D. C.; Witte M. D.; Minnaard A. J.; Bickelhaupt F. M. On the Origin of Regioselectivity in Palladium-Catalyzed Oxidation of Glucosides. Eur. J. Org. Chem. 2021, 2021, 632–636. 10.1002/ejoc.202001453.

[ref14] Eisink N. N. H. M.; Lohse J.; Witte M. D.; Minnaard A. J. Regioselective oxidation of unprotected 1,4 linked glucans. Org. Biomol. Chem. 2016, 14, 4859–4864. 10.1039/C6OB00608F. 27159790

[ref15] Jumde V. R.; Eisink N. N. H. M.; Witte M. D.; Minnaard A. J. C3 Epimerization of Glucose, via Regioselective Oxidation and Reduction. J. Org. Chem. 2016, 81, 11439–11443. 10.1021/acs.joc.6b02074. 27755870

[ref16] Eisink N. N. H. M.; Minnaard A. J.; Witte M. D. Chemo- and Regioselective Oxidation of Secondary Alcohols in Vicinal Diols. Synthesis 2017, 49, 822–829. 10.1055/s-0036-1589476.

[ref17] Zhang J.; Eisink N. N. H. M.; Witte M. D.; Minnaard A. J. Regioselective Manipulation of GlcNAc Provides Allosamine, Lividosamine, and Related Compounds. J. Org. Chem. 2019, 84, 516–525. 10.1021/acs.joc.8b01949. 30569712 PMC6343366

[ref18] Gorelik D. J.; Dimakos V.; Adrianov T.; Taylor M. S. Photocatalytic, site-selective oxidations of carbohydrates. Chem. Commun. 2021, 57, 12135–12138. 10.1039/D1CC05124E. 34723300

[ref19] Wu M.; Jiang Q.; Tian Q.; Guo T.; Cai F.; Tang S.; Liu J.; Wang X. Enzyme-like C–H Oxidation of Glucosides Promoted by Visible Light. CCS Chem. 2022, 4, 3599–3608. 10.31635/ccschem.022.202101621.

[ref20] Mouthaan M. L. M. C.; Pouwer K.; Borst M. L. G.; Witte M. D.; Minnaard A. J. α-C–H Photoalkylation of a Glucose Derivative in Continuous Flow. Synthesis 2022, 54, 4683–4689. 10.1055/a-1840-5483.

[ref21] Wan I. C. (. S.).; Witte M. D.; Minnaard A. J. Site-selective carbon–carbon bond formation in unprotected monosaccharides using photoredox catalysis. Chem. Commun. 2017, 53, 4926–4929. 10.1039/C7CC01416C. 28425550

[ref22] Wang Y.; Carder H. M.; Wendlandt A. E. Synthesis of rare sugar isomers through site-selective epimerization. Nature 2020, 578, 403–408. 10.1038/s41586-020-1937-1. 31940659

[ref23] Carder H. M.; Suh C. E.; Wendlandt A. E. A Unified Strategy to Access 2- and 4-Deoxygenated Sugars Enabled by Manganese-Promoted 1,2-Radical Migration. J. Am. Chem. Soc. 2021, 143, 13798–13805. 10.1021/jacs.1c05993. 34406756

[ref24] Carder H. M.; Wang Y.; Wendlandt A. E. Selective Axial-to-Equatorial Epimerization of Carbohydrates. J. Am. Chem. Soc. 2022, 144, 11870–11877. 10.1021/jacs.2c04743. 35731921 PMC9699703

[ref25] Oswood C. J.; MacMillan D. W. C. Selective Isomerization via Transient Thermodynamic Control: Dynamic Epimerization of trans to cis Diols. J. Am. Chem. Soc. 2022, 144, 93–98. 10.1021/jacs.1c11552. 34933555 PMC9676085

[ref26] Dimakos V.; Su H. Y.; Garrett G. E.; Taylor M. S. Site-Selective and Stereoselective C–H Alkylations of Carbohydrates via Combined Diarylborinic Acid and Photoredox Catalysis. J. Am. Chem. Soc. 2019, 141, 5149–5153. 10.1021/jacs.9b01531. 30900897

[ref27] Schnatbaum K.; Schäfer H. J. Electroorganic Synthesis 66: Selective Anodic Oxidation of Carbohydrates Mediated by TEMPO. Synthesis 1999, 1999, 864–872. 10.1055/s-1999-3464.

[ref28] Parpot P.; Servat K.; Bettencourt A. P.; Huser H.; Kokoh K. B. TEMPO mediated oxidation of carbohydrates using electrochemical methods. Cellulose 2010, 17, 815–824. 10.1007/s10570-010-9417-7.

[ref29] Vedovato V.; Vanbroekhoven K.; Pant D.; Helsen J. Electrosynthesis of Biobased Chemicals Using Carbohydrates as a Feedstock. Molecules 2020, 25, 3712 10.3390/molecules25163712. 32823995 PMC7464535

[ref30] Belgsir E. M.; Schäfer H. J. Selective oxidation of carbohydrates on Nafion®–TEMPO-modified graphite felt electrodes. Electrochem. Commun. 2001, 3, 32–35. 10.1016/S1388-2481(00)00137-5.

[ref31] Kawamata Y.; Yan M.; Liu Z.; Bao D.-H.; Chen J.; Starr J. T.; Baran P. S. Scalable, Electrochemical Oxidation of Unactivated C–H Bonds. J. Am. Chem. Soc. 2017, 139, 7448–7451. 10.1021/jacs.7b03539. 28510449 PMC5465511

[ref32] Vorobjov F.; De Smet G.; Daems N.; Vincent Ching H. Y.; Leveque P.; Maes B. U. W.; Breugelmans T. Electrochemical quinuclidine-mediated C-H activation: intermediates and mechanism. J. Electroanal. Chem. 2022, 924, 116835 10.1016/j.jelechem.2022.116835.

[ref33] Turner J. A.; Adrianov T.; Zakaria M. A.; Taylor M. S. Effects of Configuration and Substitution on C–H Bond Dissociation Enthalpies in Carbohydrate Derivatives: A Systematic Computational Study. J. Org. Chem. 2022, 87, 1421–1433. 10.1021/acs.joc.1c02725. 34964632

[ref34] Colomer I.; Chamberlain A. E. R.; Haughey M. B.; Donohoe T. J. Hexafluoroisopropanol as a highly versatile solvent. Nat. Rev. Chem. 2017, 1, 0088 10.1038/s41570-017-0088.

[ref35] Beil S. B.; Müller T.; Sillart S. B.; Franzmann P.; Bomm A.; Holtkamp M.; Karst U.; Schade W.; Waldvogel S. R. Active Molybdenum-Based Anode for Dehydrogenative Coupling Reactions. Angew. Chem., Int. Ed. 2018, 57, 2450–2454. 10.1002/anie.201712718. 29318724

[ref36] Röckl J. L.; Pollok D.; Franke R.; Waldvogel S. R. A Decade of Electrochemical Dehydrogenative C C-Coupling of Aryls. Acc. Chem. Res. 2020, 53, 45–61. 10.1021/acs.accounts.9b00511. 31850730

[ref37] Horn E. J.; Rosen B. R.; Chen Y.; Tang J.; Chen K.; Eastgate M. D.; Baran P. S. Scalable and sustainable electrochemical allylic C–H oxidation. Nature 2016, 533, 77–81. 10.1038/nature17431. 27096371 PMC4860034

[ref38] Beil S. B.; Pollok D.; Waldvogel S. R. Reproducibility in Electroorganic Synthesis—Myths and Misunderstandings. Angew. Chem., Int. Ed. 2021, 60, 14750–14759. 10.1002/anie.202014544. PMC8251947 33428811

[ref39] Chung K.; Waymouth R. M. Selective Catalytic Oxidation of Unprotected Carbohydrates. ACS Catal. 2016, 6, 4653–4659. 10.1021/acscatal.6b01501.

[ref40] Eisink N. N. H. M.; Witte M. D.; Minnaard A. J. Regioselective Carbohydrate Oxidations: A Nuclear Magnetic Resonance (NMR) Study on Selectivity, Rate, and Side-Product Formation. ACS Catal. 2017, 7, 1438–1445. 10.1021/acscatal.6b03459. 28367353 PMC5370080

[ref41] Peters B. K.; Rodriguez K. X.; Reisberg S. H.; Beil S. B.; Hickey D. P.; Kawamata Y.; Collins M.; Starr J.; Chen L.; Udyavara S.; Klunder K.; Gorey T. J.; Anderson S. L.; Neurock M.; Minteer S. D.; Baran P. S. Scalable and safe synthetic organic electroreduction inspired by Li-ion battery chemistry. Science 2019, 363, 838–845. 10.1126/science.aav5606. 30792297 PMC7001862

[ref42] Hayday K.; McKelvey R. D. An anomeric effect in photochemical hydrogen abstraction reactions of tetrahydropyranyl ethers. J. Org. Chem. 1976, 41, 2222–2223. 10.1021/jo00874a037.

[ref43] Apffel A.; Chakel J. A.; Fischer S.; Lichtenwalter K.; Hancock W. S. Analysis of Oligonucleotides by HPLC–Electrospray Ionization Mass Spectrometry. Anal. Chem. 1997, 69, 1320–1325. 10.1021/ac960916h. 21639339

[ref44] Röckl J. L.; Schollmeyer D.; Franke R.; Waldvogel S. R. Dehydrogenative Anodic C–C Coupling of Phenols Bearing Electron-Withdrawing Groups. Angew. Chem., Int. Ed. 2020, 59, 315–319. 10.1002/anie.201910077. PMC6973026 31498544

