# Supplementary material for: Site-Selective Electrochemical Oxidation of Glycosides
Source: ACS Catal. 2023 Jan 31;13(4):2335–40. doi: 10.1021/acscatal.2c06318 (PMC9942207; doi:10.1021/acscatal.2c06318)
Supplement: Supplementary file 1 — cs2c06318_si_001.pdf [file cs2c06318_si_001.pdf]

Supporting Information

Site-selective electrochemical oxidation of glycosides

Marios Kidonakis, Augustin Villotet, Martin D. Witte*, Sebastian B. Beil*, and Adriaan J. Minnaard*

Stratingh Institute for Chemistry, University of Groningen, Nijenborgh 7, 9747 AG, Groningen, The Netherlands. E-mail: M.D.Witte@rug.nl, S.B.Beil@rug.nl, A.J.Minnaard@rug.nl

Table of Contents

General Information S2

Starting materials that were synthesized: S2

General Procedure for the Regioselective Electrochemical Oxidation S4

Procedure for the Large Scale Oxidation S5

Data of the Products S6

Investigations into other HAT agents S13

Alternative mechanism S14

Kinetics of the influence of quinuclidine loading S15

Competition experiments with electro-rich arenes S16

Cyclic voltammetry investigations S17

Literature Comparison with Sclareolide Oxidation S18

^{1}H, ^{13}C –NMR and HRMS Spectra of the New Compounds S19

General Information

All solvents used were of commercial grade and used without further purification. Quinuclidine, Me_4NBF_4 and 1,1,1,3,3,3-hexafluoro-2-propanol were purchased from Sigma-Aldrich and TCI and were used without further purification. Electrochemical reactions were performed in an ElectraSyn 2.0 device with IKA graphite electrodes. Large-scale oxidation was performed with a VSP-300 (BioLogic) potentiostat using graphite electrodes (SIGRAFINE®, SGL Carbon SE). Flash chromatography was performed manually with silica gel (SiliaFlash P60, 230-400 mesh, Silicycle) or performed with automated column chromatography using a Reveleris flash chromatography system purchased from Büchi. TLC was performed on Merck silica gel 60, 0.25 mm plates and visualization was done by staining with anisaldehyde stain. NMR spectra were recorded on a Varian AMX400 spectrometer using CD_3OD, CD_3CN or $CDCl_3$. Data are reported as follows: Chemical shifts (δ), multiplicity (s = singlet, d = doublet, m = multiplet, br = broad), coupling constants J (Hz), and integration. High-resolution mass spectra (HRMS) were recorded on a Thermo Scientific LTQ Orbitrap XL.

Starting materials that were synthesized:

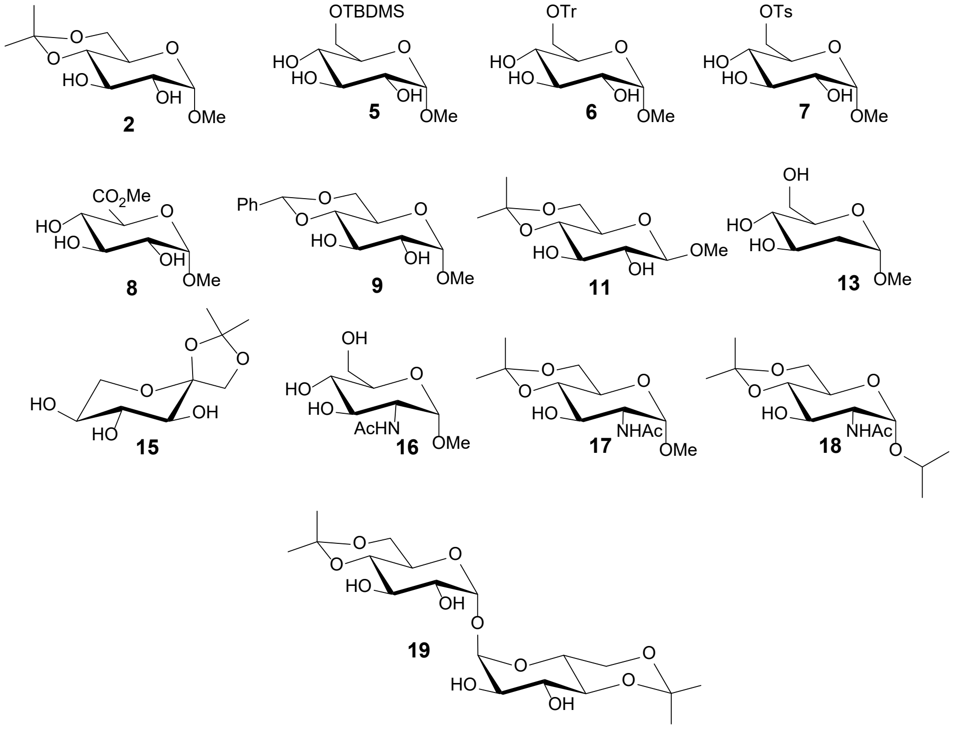

Compounds **1**, **10, 12**, **14** are commercially available. Compounds **2**,[1] **5**,[3] **6**,**Error! Reference source not found.** **7**,[4] **8**,[7] **9**,[6] **11**,[1] **13**,[7] **15**,[8] **16**,[9] **17**,[10] and **19**[11] were synthesized according to known literature procedures and the characterization data are in full agreement.

Synthesis of compound **18**.

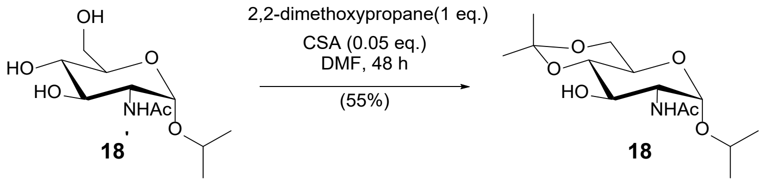

To a stirred solution of compound **18’** (0.5 gr, 1.90 mmol) in 10 mL DMF were added (*S*)-camphorsulfonic acid (0.01 eq.) and 2,2-dimethoxypropane (1 eq.). The resulting mixture was stirred at room temperature for 48 h, upon which water and EtOAc were added. The resulting phases were separated and the aqueous layer was extracted with EtOAc (3x). The combined organic layers were washed with water (3x), dried over $MgSO_4$ and evaporated under reduced pressure. Compound **18** was isolated upon column chromatography (10/1 DCM : MeOH) as a white solid (319mgr, 55%).

^{1}H-NMR (400 MHz, CD_3OD) δ 4.89 (d, J = 3.9 Hz, 1H), 3.94 (dd, J = 10.3, 3.9 Hz, 1H), 3.83 (p, J = 6.2 Hz, 1H), 3.80-3.74 (m, 2H), 3.73-3.64 (m, 2H), 3.64-3.53 (m, 1H), 1.98 (s, 3H), 1.52 (s, 3H), 1.47 (s, 3H), 1.22 (d, J = 6.3 Hz, 3H), 1.13 (d, J = 6.1 Hz, 3H).

^{13}C-NMR (101 MHz, CD_3OD) δ 173.6, 100.9, 97.7, 76.3, 71.8, 69.9, 65.1, 63.4, 56.0, 29.5, 23.7, 22.5, 21.8, 19.4.

HRMS (ESI^-) calculated for $C_{14}H_{24}N_1O_6$ ($[M-H]^-$): 302.16091, found: 302.16046.

General Procedure for the Regioselective Electrochemical Oxidation

A mixture of starting material (1 equiv.), quinuclidine (0.3 equiv.), Me_4NBF_4 (1 equiv.) and HFIP (10 equiv.) in CH_3CN (5 mL) was placed in an ElectraSyn vial, equipped with two IKA graphite electrodes, and electrolyzed in a Electrasyn 2.0 at room temperature, under constant current (5 mA, j = 2.00 mA/cm^2). The reaction progress was monitored by TLC (10% MeOH in DCM). Acetonitrile and HFIP were subsequently co-evaporated with water under reduced pressure, upon which celite and MeOH were added and the slurry was concentrated to dryness at 40 ºC. The resulting mixture was pulverized, loaded on top of a silica gel column and subsequently eluted (5 to 10% MeOH in DCM). Fractions containing the desired product were collected and the solvent was removed in vacuo.

Procedure for the Large Scale Oxidation

To a 30 mL beaker-type electrolysis cell (Scheme 1) were added 1 g of glycoside **5** (3.24 mmol, 1.0 eq.), quinuclidine (0.973 mmol, 0.3 eq.), Me_4NBF_4 (3.24 mmol, 1.0 eq.), HFIP (5.45 mmol, 10 eq.) and MeCN (25 mL). Graphite electrodes (2.0 cm x 6.0 cm) were immersed in the solution and electrolysis was conducted under constant current (5 mA). Electrolysis was conducted for 96 h, upon which the mixture was transferred into a round-bottom flask. MeCN and HFIP were evaporated under reduced pressure, celite and MeOH were added and the slurry was concentrated to dryness at 40 ºC. The resulting mixture was pulverized, loaded on a silica gel column and eluted (5 % MeOH in DCM). The fractions containing the product were combined and the solvent was evaporated in vacuo. Compound **5a** was isolated as a white solid (469 mg, 1.53 mmol, 50%).

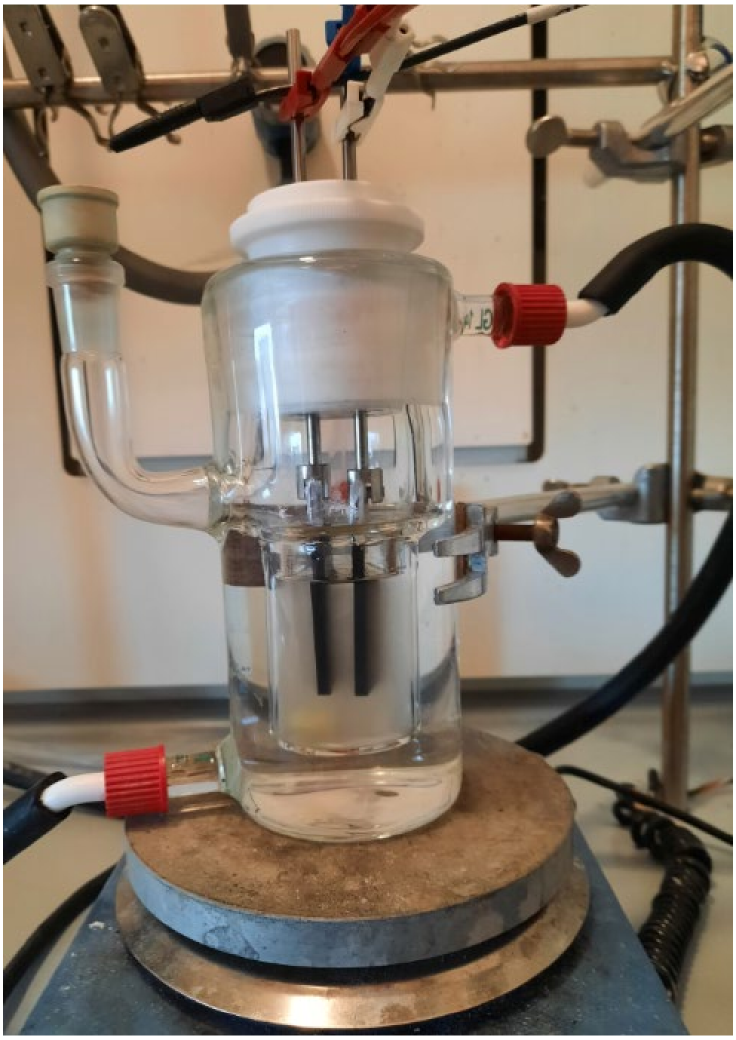

Figure S1. Gram-scale electrochemical oxidation.

Data of the Products

(2*R*,3*R*,5*S*,6*S*)-3,5-Dihydroxy-2-(hydroxymethyl)-6-methoxytetrahydro-4*H*-pyran-4-one (**1a**).

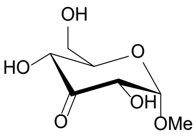

100 mg (0.52 mmol) of compound **1** were used. Isolated as a white solid (55 mg, 0.29 mmol, 56%) after column chromatography (10% MeOH in DCM).

^{1}H NMR (400 MHz, CD_3OD) δ 5.05 (d, *J* = 4.2 Hz, 1H), 4.40 (dd, *J* = 4.3, 1.5 Hz, 1H), 4.23 (dd, *J* = 9.7, 1.6 Hz, 1H), 3.88 (dd, *J* = 12.1, 2.2 Hz, 1H), ,3.83-3.73 (m, 1H) 3.65 (ddd, J = 9.8, 4.7, 2.2 Hz, 1H), 3.40 (s, 3H).

^{13}C NMR (101 MHz, CD_3OD) δ 207.0, 103.8, 76.7, 76.1, 73.3, 62.5, 55.7.

Spectroscopic data correspond to those reported in literature.[12]

(4*aR*,6*S*,7*S*,8*aR*)-7-Hydroxy-6-methoxy-2,2-dimethyltetrahydropyrano[3,2-d][1,3]dioxin-8(4*H*)-one (**2a**).

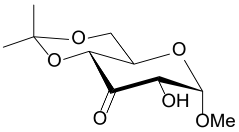

50 mg (0.21 mmol) of compound **2** were used. Isolated as a white solid (29.6 mg, 0.13 mmol, 60%) after column chromatography (5% MeOH in DCM).

^{1}H NMR (400 MHz, CD_3CN) δ 5.08 (d, *J* = 4.3 Hz, 1H), 4.49 (dd, *J* = 10.0, 1.6 Hz, 1H), 4.34 (ddd, *J* = 8.4, 4.2, 1.5 Hz, 1H), 3.92 (m, 2H), 3.72 (td, *J* = 9.5, 6.4 Hz, 1H), 3.51 (d, *J* = 8.8 Hz, 1H), 3.36 (s, 3H), 1.48 (s, 3H), 1.38 (s, 3H).

^{13}C NMR (101 MHz, CD_3CN) δ 201.1, 104.6, 100.8, 76.2, 76.0, 67.6, 63.4, 56.0, 29.1, 19.3.

Spectroscopic data correspond to those reported in literature.[13]

(2*R*,3*R*,5*S*,6*S*)-2-(((tert-butyldimethylsilyl)oxy)methyl)-3,5-dihydroxy-6-methoxytetrahydro-4*H*-pyran-4-one (**5a**).

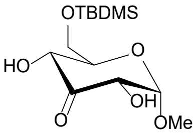

100 mg (0.32 mmol) of compound **5** were used. Isolated as a white solid (62 mg, 0.20 mmol, 62%) after column chromatography (5% MeOH in DCM).

^{1}H NMR (400 MHz, CD_3CN) δ 5.03 (d, *J* = 4.3 Hz, 1H), 4.34 (dd, *J* = 4.3, 1.6 Hz, 1H), 4.23 (dd, *J* = 9.7, 1.6 Hz, 1H), 4.03-3.81 (m, 2H), 3.54 (ddd, *J* = 9.7, 4.2, 2.1 Hz, 1H), 3.34 (s, 3H), 0.92 (s, 9H), 0.10 (s, 6H).

^{13}C NMR (101 MHz, CD_3CN) δ 207.3, 103.2, 76.6, 75.8, 72.9, 63.3, 55.7, 28.8, 26.2, 19.0, -5.1, -5.2.

Spectroscopic data correspond to those reported in literature.[13]

(2*S*,3*S*,5*R*,6*R*)-3,5-Dihydroxy-2-methoxy-6-((trityloxy)methyl)tetrahydro-4*H*-pyran-4-one (**6a**).

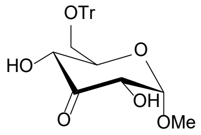

170 mg (0.39 mmol) of compound **6** were used. Reaction was run on 0.46 mmol scale. Isolated as white solid (118 mg, 0.27 mmol, 70%) after column chromatography (5% MeOH in DCM).13

^{1}H NMR (400 MHz, CD_3CN) δ 7.52-7.46 (m, 6H), 7.37-7.31 (m, 6H), 7.30-7.24 (m, 3H), 5.11 (d, *J* = 4.3 Hz, 1H), 4.50-4.38 (m, 1H), 4.28 (d, *J* = 9.8, 5.1, 1.5 Hz, 1H), 3.77-3.72 (m, 1H), 3.62 (d, *J* = 5.3 Hz, 1H), 3.57(d, *J* = 7.9 Hz, 1H), 3.42 (s, 3H), 3.40-3.28 (m, 2H).

^{13}C NMR (101 MHz, CD_3CN) δ 206.8, 145.0, 129.6, 128.9, 128.1, 103.2, 87.3, 75.9, 75.3, 73.6, 64.4, 55.8.

Spectroscopic data correspond to those reported in literature.[13]

((2*R*,3*R*,5*S*,6*S*)-3,5-Dihydroxy-6-methoxy-4-oxotetrahydro-2*H*-pyran-2-yl)methyl 4-methylbenzenesulfonate (**7a**).

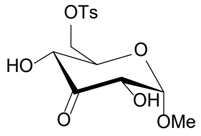

200 mg (0.57 mmol) of compound **7** were used. Reaction was run on 0.57 mmol scale. Isolated as a yellow sticky oil (105 mg, 0.30 mmol, 53%) after column chromatography (3% MeOH in DCM).

^{1}H NMR (400 MHz, CD_3OD) δ 7.82 (d, *J* = 8.35 Hz, 2H), 7.45 (d, *J* = 8.0 Hz, 2H), 4.94 (d, *J* = 4.2 Hz, 1H), 4.37 (dd, *J* = 11.0, 2.1 Hz, 1H), 4.34 (dd, *J* = 4.3, 1.5 Hz, 1H), 4.30 (dd, *J* = 11.0, 5.3 Hz, 1H), 4.11 (dd, *J* = 10.0, 1.5 Hz, 1H), 3.77 (ddd, *J* = 10.0, 5.5, 2.1 Hz, 1H), 3.34 (s, 3H), 2.45 (s, 3H).

^{13}C NMR (101 MHz, CD_3OD) δ 205.8, 146.6, 134.3, 131.1, 129.1, 103.6, 75.9, 73.7, 73.1, 70.6, 55.9, 21.6.

HRMS (ESI+) calculated for $C_{14}H_{18}O_8S_1Na_1$ ([M+Na]+): 369.0615, found: 369.0608.

Methyl (2*S*,3*R*,5*S*,6*S*)-3,5-Dihydroxy-6-methoxy-4-oxotetrahydro-2*H*-pyran-2-carboxylate (**8a**).

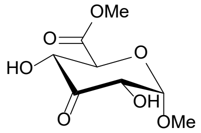

50 mg (0.23 mmol) of compound **8** were used. Isolated as a white solid (24.3 mg, 0.11 mmol, 49%) after column chromatography (10% MeOH in DCM).

^{1}H NMR (400 MHz, CD_3CN) δ 5.11 (d, *J* = 4.1 Hz, 1H), 4.48-4.39 (m, 2H), 4.06 (dd, *J* = 9.7, 0.6 Hz, 1H), 3.79 (s, 3H), 3.39 (s, 3H).

^{13}C NMR (101 MHz, CD_3CN) δ 205.0, 169.8, 103.9, 75.8, 74.7, 74.6, 56.4, 53.3.

Spectroscopic data correspond to those reported in literature.[5]

(2*R*,4a*R*,6*S*,7*S*,8a*R*)-7-hydroxy-6-methoxy-2-phenyltetrahydropyrano[3,2-d][1,3]dioxin-8(4*H*)-one (**9a**).

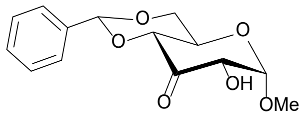

100 mg (0.35 mmol) of compound **9** were used. Isolated as a white solid (59.8 mg, 0.21 mmol, 60%) after column chromatography (5% MeOH in DCM).

^{1}H NMR (400 MHz, $CDCl_3$) δ 7.52-7.50 (m, 2H), 7.38-7.36 (m, 3H), 5.58 (s, 1H), 5.17 (d, *J* = 4.4 Hz, 1H), 4.44 – 4.39 (m, 2H), 4.35 (dd, *J* = 9.5, 1.6 Hz, 1H), 4.06 (td, *J* = 9.8, 4.5 Hz, 1H), 3.95 (t, *J* = 10.2 Hz, 1H), 3.46 (s, 3H).

^{13}C NMR (101 MHz, $CDCl_3$) δ 198.4, 136.4, 129.6, 128.5, 126.5, 103.5, 102.2, 82.2, 75.2, 69.7, 66.1, 56.0.

Spectroscopic data correspond to those reported in literature.[14]

(4a*R*,6*R*,7*S*,8a*R*)-7-Hydroxy-6-methoxy-2,2-dimethyltetrahydropyrano[3,2-d][1,3]dioxin-8(4*H*)-one (**11a**).

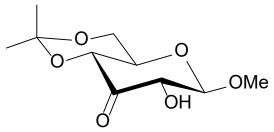

50 mg (0.21 mmol) of compound **11** were used. Isolated as white solid (10.3 mg, 0.05 mmol, 21%) after column chromatography (5% MeOH in DCM).

^{1}H NMR (400 MHz, $CDCl_3$) δ 4.44 (dd, *J* = 10.2, 1.9 Hz, 1H), 4.35 (d, *J* = 7.4 Hz, 1H), 4.13 (dd, *J* = 7.5, 1.9 Hz, 1H), 4.09 (dd, *J* = 11.0, 5.2 Hz, 1H), 3.96 (t, *J* = 10.4 Hz, 1H), 3.63 (s, 3H), 3.41 (ddd, *J* = 15.3, 10.2, 5.2 Hz, 1H), 1.53 (s, 3H), 1.51 (s, 3H).

^{13}C NMR (101 MHz, $CDCl_3$) δ 199.8, 107.2, 100.7, 77.4, 75.4, 68.0, 62.6, 57.9, 28.8, 18.9.

Spectroscopic data correspond to those reported in literature.[13]

(2*S*,3*S*,5*R*)-3,5-Dihydroxy-2-methoxytetrahydro-4*H*-pyran-4-one (**12a**).

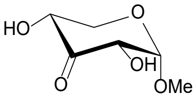

50 mg (0.31 mmol) of compound **12** were used. Isolated as a white solid (20.2 mg, 0.12 mmol, 41%) after column chromatography (5% MeOH in DCM).

^{1}H NMR (400 MHz, CD_3OD) δ 4.97 (d, *J* = 4.2 Hz, 1H), 4.41-4.34 (m, 2H), 3.97 (dd, *J* = 10.3, 7.9 Hz, 1H), 3.58 (t, *J* = 10.4 Hz, 1H), 3.35 (s, 3H).

^{13}C NMR (101 MHz, CD_3OD) δ 206.6, 104.4, 76.3, 73.1, 65.2, 55.7.

Spectroscopic data correspond to those reported in literature.[13]

(2*R*,3*R*,6*S*)-3-Hydroxy-2-(hydroxymethyl)-6-methoxytetrahydro-4*H*-pyran-4-one (**13a**).

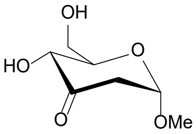

100 mg (0.56 mmol) of compound **13** were used. Isolated as a white solid (48 mg, 0.27 mmol, 49%) after column chromatography (10% MeOH in DCM).

^{1}H NMR (400 MHz, CD_3OD) δ 5.13 (d, *J* = 4.2 Hz, 1H), 4.17 (d, *J* = 9.9 Hz, 1H), 3.87 (dd, *J* = 12.0, 2.3 Hz, 1H), 3.80 (dd, *J* = 12.0, 4.7 Hz, 1H), 3.68 (ddd, *J* = 9.8, 4.6, 2.2 Hz, 1H), 3.34 (s, 2H), 2.87 (ddd, *J* = 14.1, 4.5, 1.0 Hz, 1H), 2.49 (d, *J* = 14.1 Hz, 1H).

^{13}C NMR (101 MHz, CD_3OD) δ 207.3, 101.2, 76.4, 74.1, 62.6, 55.0, 46.6.

Spectroscopic data correspond to those reported in literature.[13]

(2*S*,3*S*,5*R*,6*R*)-3,5-dihydroxy-2-methoxy-6-methyltetrahydro-4*H*-pyran-4-one (**14a**).

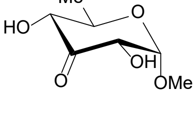

100 mg (0.56 mmol) of compound **14** were used. Isolated as a colorless sticky oil (60.2 mg, 0.34 mmol, 61%) after column chromatography (5% MeOH in DCM).

^{1}H NMR (400 MHz, CD_3CN) δ 4.98 (d, *J* = 4.4 Hz, 1H), 4.37 (ddd, *J* = 8.2, 4.2, 1.6 Hz, 1H), 3.86 (ddd, *J* = 9.4, 5.0, 1.7 Hz, 1H), 3.68-3.57 (m, 2H), 3.49 (br d, *J* = 8.0 Hz, 1H, OH), 3.34 (s, 3H), 1.36 (d, *J* = 6.2 Hz, 3H).

The spectrum was also recorded in CD_3OD.

^{1}H NMR (400 MHz, CD_3OD) δ 4.98 (d, *J* = 4.3 Hz, 1H), 4.40 (dd, *J* = 4.4, 1.7 Hz, 1H), 3.88 (dd, *J* = 9.4, 1.5 Hz, 1H), 3.71 (dq, *J* = 9.4, 6.2 Hz, 1H), 3.38 (s, 3H), 1.39 (d, *J* = 6.1 Hz, 3H).

^{13}C NMR (101 MHz, CD_3CN) δ 206.6, 103.1, 78.5, 75.8, 72.0, 55.7, 18.9.

Spectroscopic data correspond to those reported in literature.[13]

(5*S*,8*S*,10*S*)-8,10-Dihydroxy-2,2-dimethyl-1,3,6-trioxaspiro[4.5]decan-9-one (**15a**).

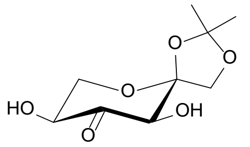

45 mg (0.20 mmol) of compound **15** were used. Isolated as colorless sticky oil (15.3 mg, 0.07 mmol, 34%) after column chromatography (5% MeOH in DCM).

^{1}H NMR (400 MHz, CD_3OD) δ 4.43 (dd, *J* = 10.6, 7.9, 1H), 4.32 (d, *J* = 1.5 Hz, 1H), 4.19 (d, *J* = 8.8 Hz, 1H), 4.08-4.03 (m, 2H), 3.70 (t, *J* = 10.5 Hz, 1H), 1.45 (s, 3H), 1.37 (s, 3H).

^{13}C NMR (101 MHz, CD_3OD) δ 206.2, 114.0, 109.9, 75.1, 73.1, 66.2, 27.3, 26.1.

HRMS (ESI^-) calculated for $C_9H_{13}O_6$ ($[M-H]^-$): 217.0718, found: 217.0716.

N-((4a*R*,6*S*,7*S*,8a*R*)-6-Methoxy-2,2-dimethyl-8-oxohexahydropyrano[3,2-d][1,3]dioxin-7-yl)acetamide (**17a**).

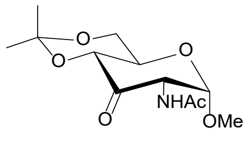

50 mg (0.18 mmol) of compound **17** were used. Isolated as colorless sticky oil (33.5 mg, 0.12 mmol, 67%) after column chromatography (5% MeOH in DCM).

^{1}H NMR (400 MHz, CD_3CN) δ 6.67 (d, *J* = 8.4 Hz, 1H), 5.08 (d, *J* = 4.2 Hz, 1H), 4.86 (ddd, *J* = 8.3, 4.2, 1.4 Hz, 1H), 4.61 (dd, *J* = 10.1, 1.4 Hz, 1H), 4.19-3.83 (m, 2H), 3.76 (td, *J* = 10.0, 5.5 Hz, 1H), 3.34 (s, 3H), 1.95 (s, 3H), 1.48 (s, 3H), 1.38 (s, 3H).

^{13}C NMR (101 MHz, CD_3CN) δ 197.9, 171.0, 103.1, 101.0, 76.7, 67.8, 63.3, 59.9, 56.0, 29.2, 22.7, 19.3.

HRMS (ESI^-) calculated for $C_{12}H_{18}NO_6$ ($[M-H]^-$): 272.1140, found: 272.1140.

N-((4a*R*,6*S*,7*S*,8a*R*)-6-isopropoxy-2,2-dimethyl-8-oxohexahydropyrano[3,2-d][1,3]dioxin-7-yl)acetamide (**18a**).

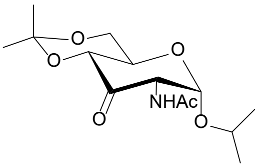

100 mg (0.33 mmol) of compound **18** were used. Isolated as sticky yellow oil (32.8 mg, 0.11 mmol, 33%) after column chromatography (5% MeOH in DCM).

^{1}H NMR (400 MHz, CD_3OD) δ 5.36 (d, J = 4.4 Hz, 1H), 4.96 (dd, J = 4.5, 1.4 Hz, 1H), 4.66 (dd, J = 9.6, 1.5 Hz, 1H), 4.08-3.97 (m, 1H), 3.96-3.84 (m, 3H), 2.03 (s, 3H), 1.53 (s, 3H), 1.42 (s, 3H), 1.21 (d, J = 6.2 Hz, 3H), 1.14 (d, J = 6.1 Hz, 3H).

^{13}C NMR (101 MHz, CD_3OD) δ 198.6, 173.4, 101.6, 100.8, 77.1, 72.4, 68.3, 63.7, 60.4, 29.1, 23.4, 22.2, 21.5, 19.3.

HRMS (ESI^-) calculated for $C_{14}H_{22}NO_6$ ($[M-H]^-$): 300.1453, found: 300.1447.

(4a*R*,4a'*R*,6*R*,6'*R*,7*S*,7'*S*,8a*R*,8a'*R*)-6,6'-Oxybis(7-hydroxy-2,2-dimethyltetrahydropyrano[3,2-d][1,3]dioxin-8(4*H*)-one) (**19a**).

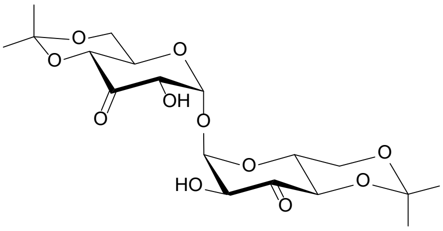

50 mg (0.12 mmol) of compound **19** were used. Isolated as white solid (21.4 mg, 0.05 mmol, 42%) after column chromatography (5% MeOH in DCM).

^{1}H NMR (400 MHz, $CDCl_3$) δ 5.50 (d, J = 4.6, 2H), 4.55-4.34 (m, 4H), 4.10 (td, J = 10.1, 5.0 Hz, 2H), 3.99 (dd, J = 10.6, 5.1 Hz, 2H), 3.91 (t, J = 10.4 Hz, 2H), 1.51 (s, 6H), 1.51 (s, 6H).

^{13}C NMR (101 MHz, $CDCl_3$) δ 199.0, 100.7, 98.4, 75.5, 74.1, 67.7, 62.7, 28.7, 19.0

Spectroscopic data correspond to those reported in literature.[13]

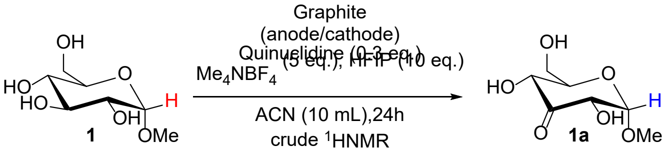

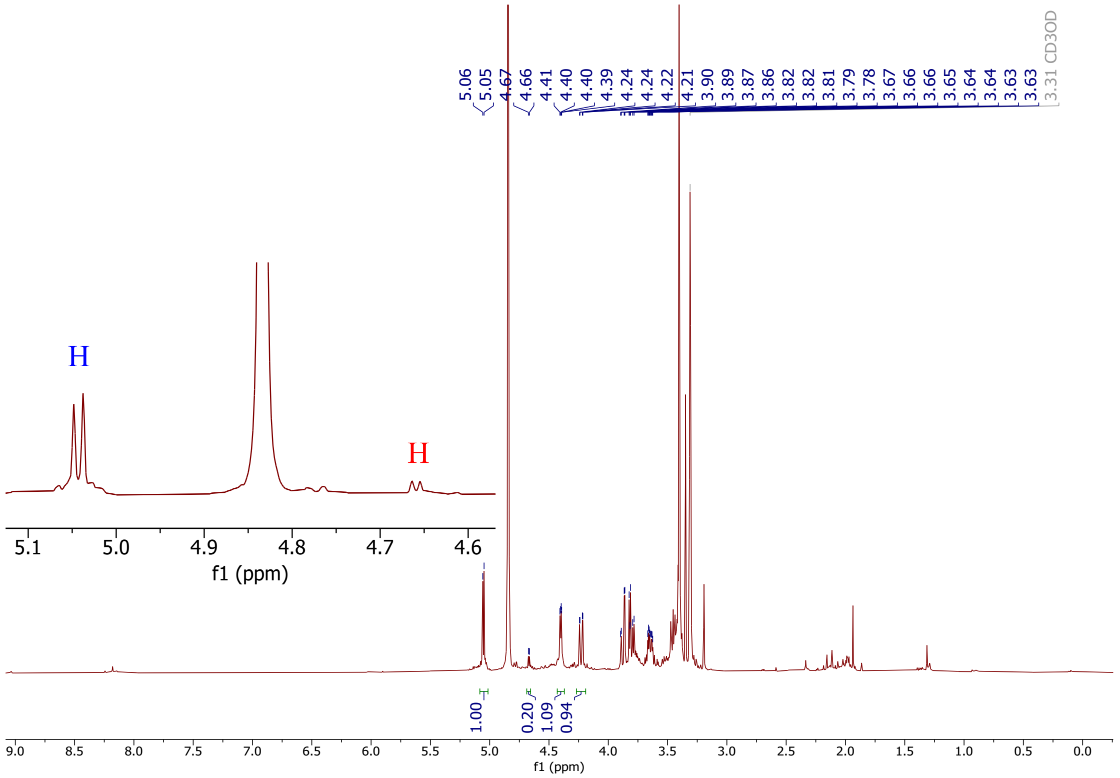

Figure S2. Crude ^{1}H NMR for the oxidation of Me-α-D-glucopyranoside.

Investigations into other HAT agents

Initially we started with the RVC || Ni system and investigated several HAT-active mediators. If no selectivity was observed, the substrate was fully consumed and no product was detected.

Table S1: Screening of HAT mediators.

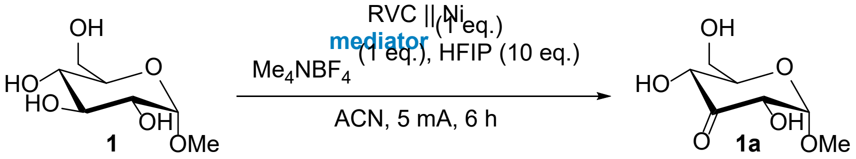

Entry	Mediator	Selectivity
1	quinuclidine	>99%
2	3-quinuclidinol	0%
3	DABCO	29%
4	DIPEA	0%
5	NHPI	0%
6	TEMPO	0%*

*complex mixture, no product detected

Alternative plausible mechanism

Alternatively to the mechanism described in Figure 2A, it is plausible that radical intermediate **I** is deprotonated to form an alkoxy intermediate which is followed by single-electron oxidation. Radical recombination would then lead to the final ketone.

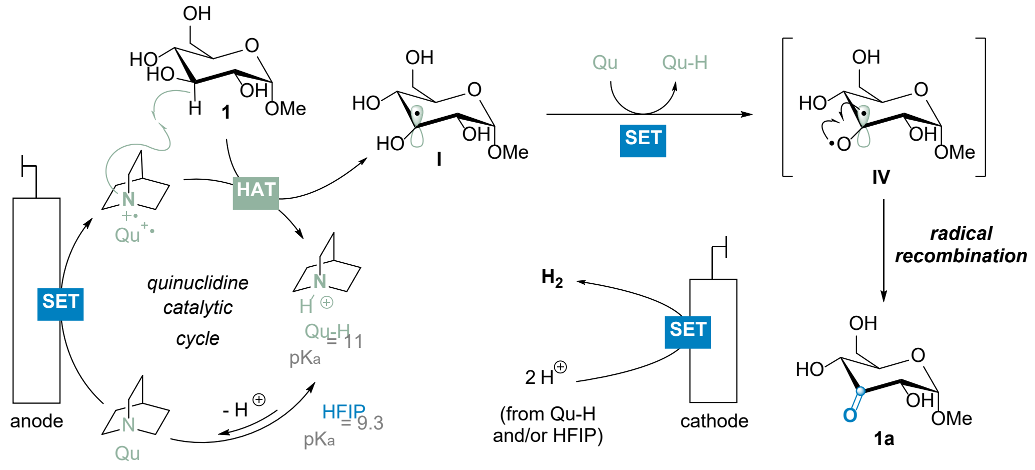

Figure S3. Alternative plausible mechanism.

Kinetics of the influence of quinuclidine loading

Kinetics obtained from ^{1}H NMR analysis of aliquots from the reaction mixture. 1,4-Difluorobenzene was used as internal standard in $CDCl_3$ as NMR solvent. NMR analysis was conduct with MestReNova 14, using whittaker smoother baseline correction.

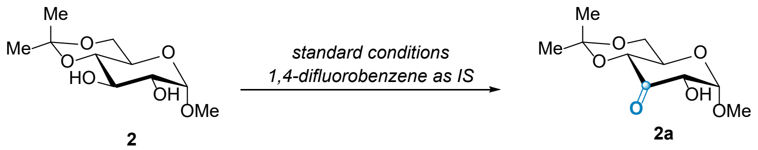

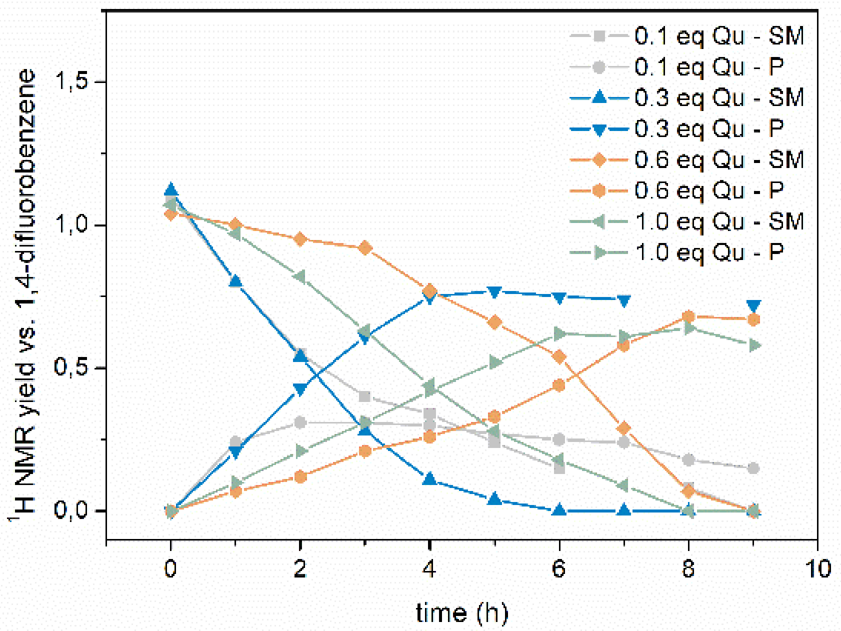

Figure S4. Reaction kinetics with 0.1 eq. (grey), 0.3 eq. (blue), 0.6 eq. (orange) and 1.0 eq. (green) of quinuclidine.

Competition experiments with electro-rich arenes

Competition experiments between glycoside **2** and 4-methyl veratrole (**3**). Under the standard reaction conditions (0.3 eq. of Qu, in blue in Figure S4) the benzylic position of the veratrole was selectively oxidized, yielding aldehyde **3b** in 23% yield, while compound **2** remained intact (recovery 89%). In the absence of quinuclidine (in green) the oxidative coupling product **3a** was formed, in 57% yield. In the latter case, compound **2** was deprotected to methyl-a-D-glycopyranoside **1** in 78% yield.

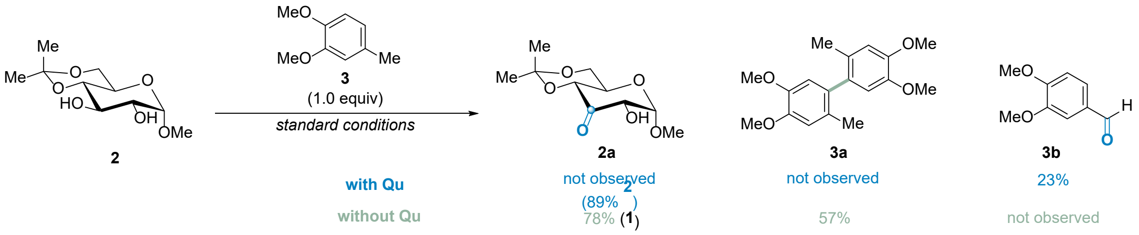

Figure S5. Competition experiment of glycoside **2** and 4-methyl veratrole (**3**) in the presence (blue) and absence (green) of quinuclidine.

Since the benzylic position caused problems under standard conditions being easily oxidized, bromo veratrole **4** was subjected in the electrolysis (compare Figure 2 in the manuscript). Under standard conditions, oxidative dimerization to product **4a** took place in 17% NMR yield (trimethoxy benzene was used as internal standard) together with recovery of 68% **4**, giving full mass balance.

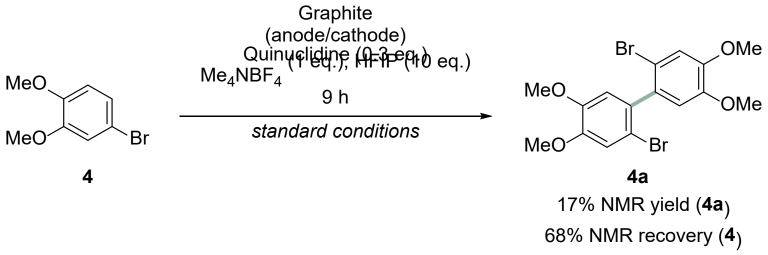

Figure S6. Dimerization of bromo veratrole (**4**) under standard reaction conditions.

Cyclic voltammetry investigations

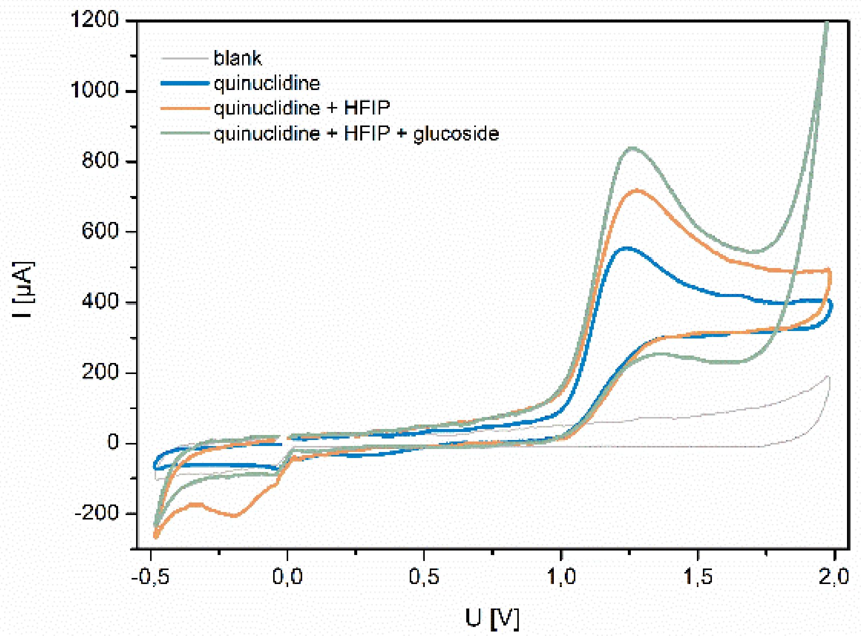

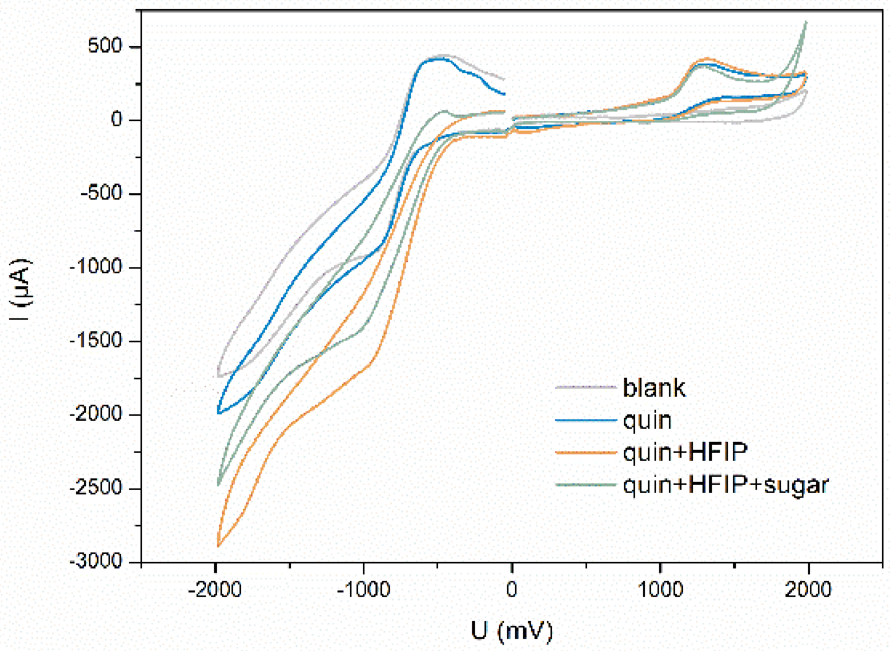

Figure S7. Cyclic voltammograms of 1) 3 mM quinuclidine (blue line), subsequently added 2) 100 mM HFIP (orange line) and 3) 10 mM **5** (green line), working electrode: glassy carbon, counter electrode: Pt (top) or GC (bottom), reference electrode: Ag/AgCl, electrolyte: 50 mM NMe_4BF_4 in MeCN (gray line), scan rate: 100 mV s^{-1}.

Literature Comparison with Sclareolide Oxidation

Oxidation of sclareolide was conducted as described by Baran et. al. (ref. 13 in the manuscript).

A

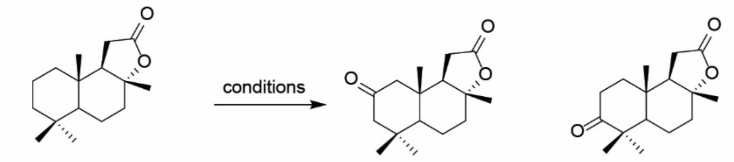

B starting material

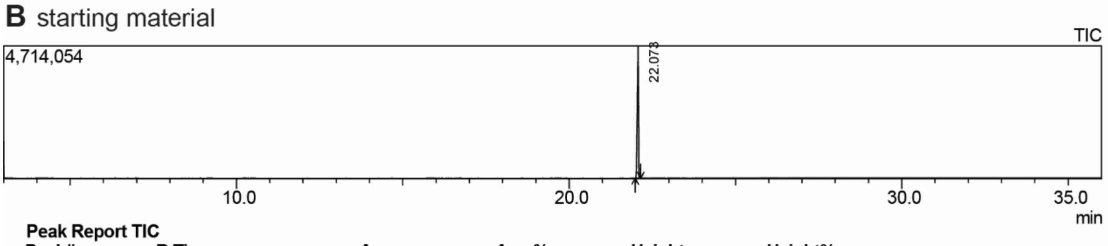

Peak Report TIC

Peak#	R.Time	Area	Area%	Height	Height%
sclareolide	22.073	12362567	100.00	4686960	100.00
Total		12362567	100.00	4686960	100.00

C reaction under oxygen

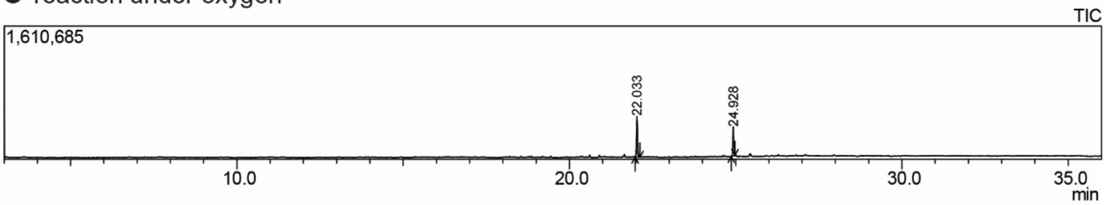

Peak Report TIC

Peak#	R.Time	Area	Area%	Height	Height%
sclareolide	22.033	998668	59.57	491149	58.37
oxo-sclareolide	24.928	677798	40.43	350231	41.63
Total		1676466	100.00	841380	100.00

D reaction under oxygen free conditions

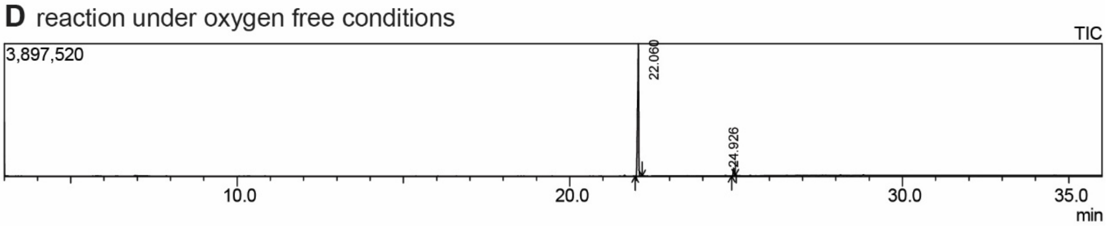

Peak Report TIC

Peak#	R.Time	Area	Area%	Height	Height%
sclareolide	22.060	9382731	95.69	3879238	94.68
oxo-sclareolide	24.926	422256	4.31	218069	5.32
Total		9804987	100.00	4097307	100.00

Figure S8. Control reaction on sclareolide. (A) representation of the reaction. (B) GCMS trace of the starting material, (C) GCMS trace of the reaction performed in the presence of oxygen, (D) GCMS trace of the reaction performed under oxygen free conditions.

^{1}H, ^{13}C –NMR and HRMS Spectra of the New Compounds

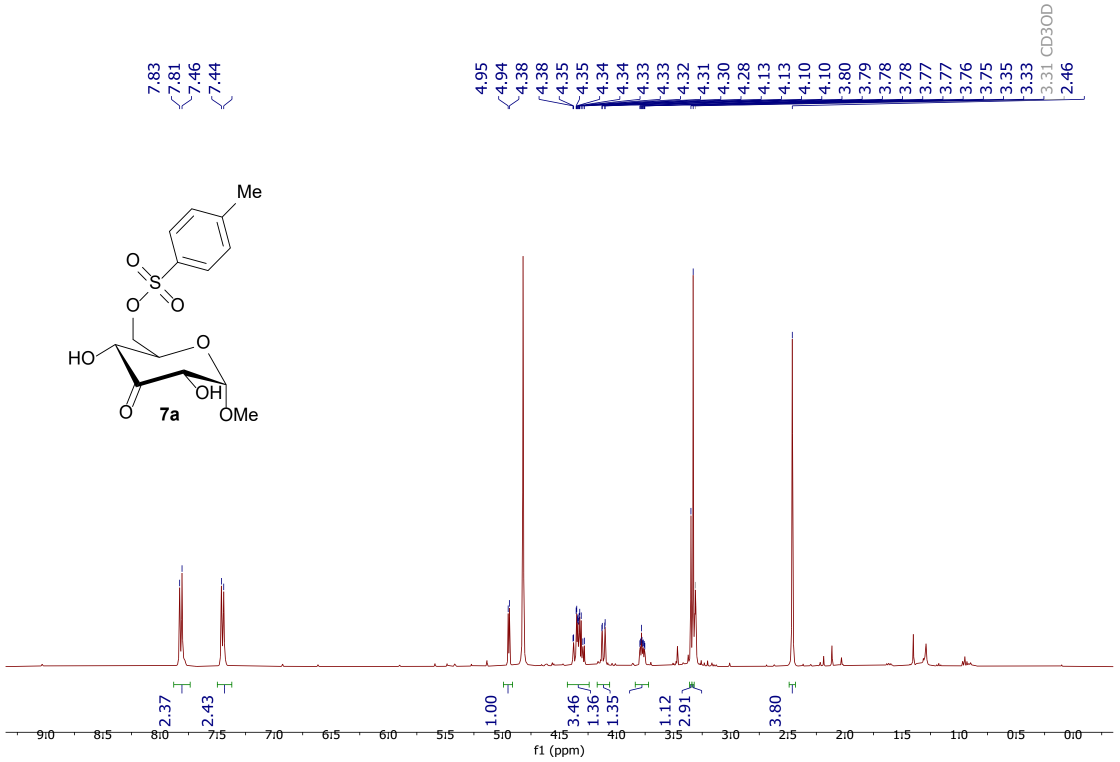

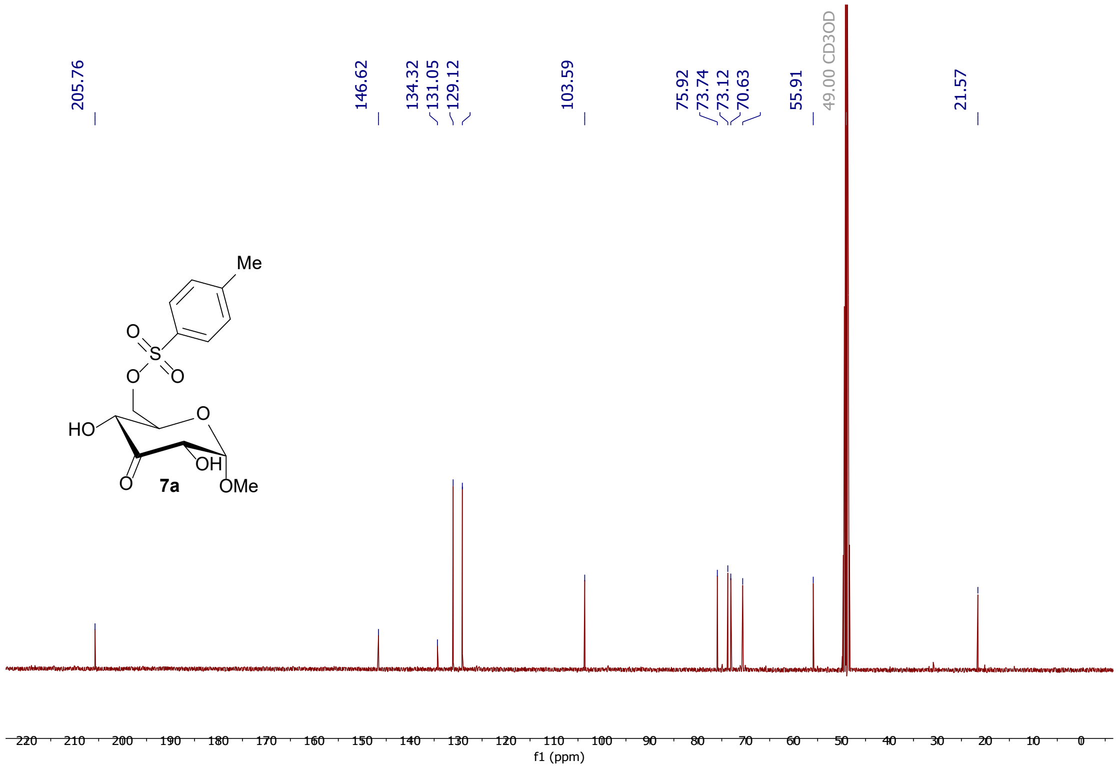

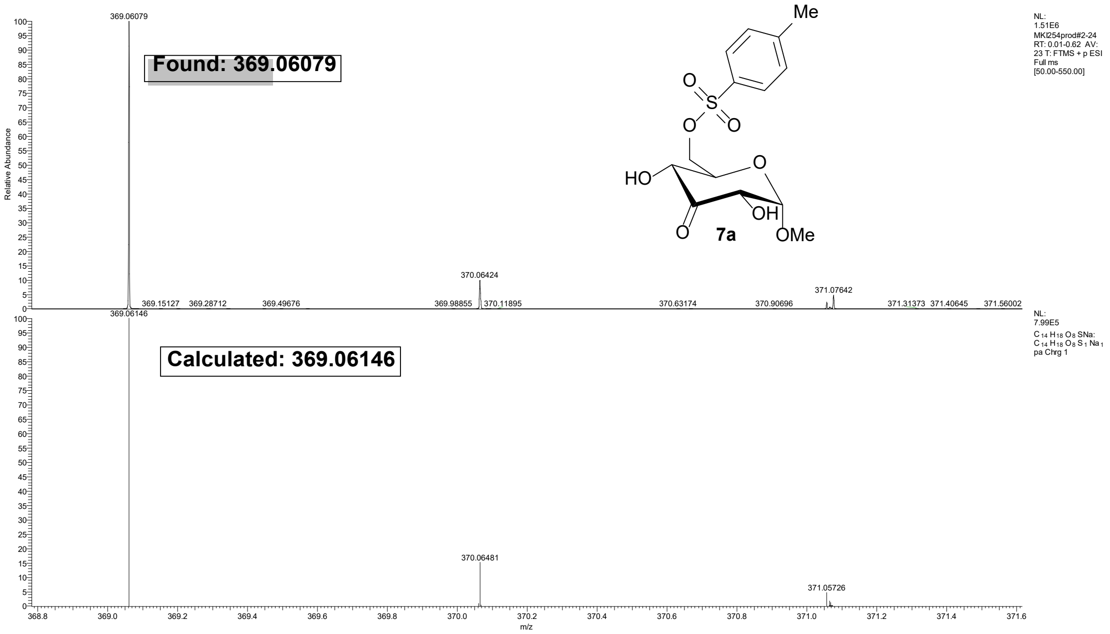
Found: 369.06079
Calculated: 369.06146
7a
NL: 1.51E6
MKI254prod#2-24
RT: 0.01-0.62 AV: 23 T: FTMS + p ESI Full ms [50.00-550.00]
NL: 7.99E5
C 14 H 18 O 8 SNa:
C 14 H 18 O 8 S 1 Na 1
pa Chrg 1
Relative Abundance
m/z

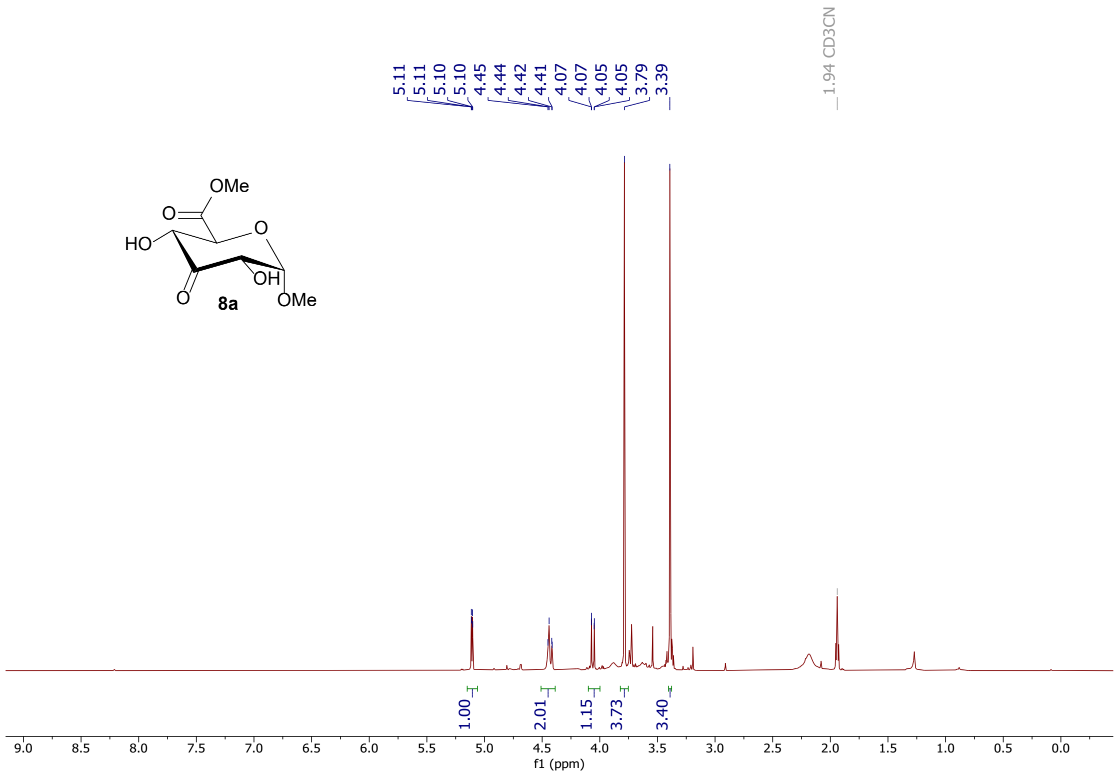
5.11
5.11
5.10
5.10
4.45
4.44
4.42
4.41
4.07
4.07
4.05
4.05
3.79
3.39
1.94 CD3CN
OMe
O
HO
O
OH
O
8a
OMe
1.00
2.01
1.15
3.73
3.40
9.0 8.5 8.0 7.5 7.0 6.5 6.0 5.5 5.0 4.5 4.0 3.5 3.0 2.5 2.0 1.5 1.0 0.5 0.0
f1 (ppm)

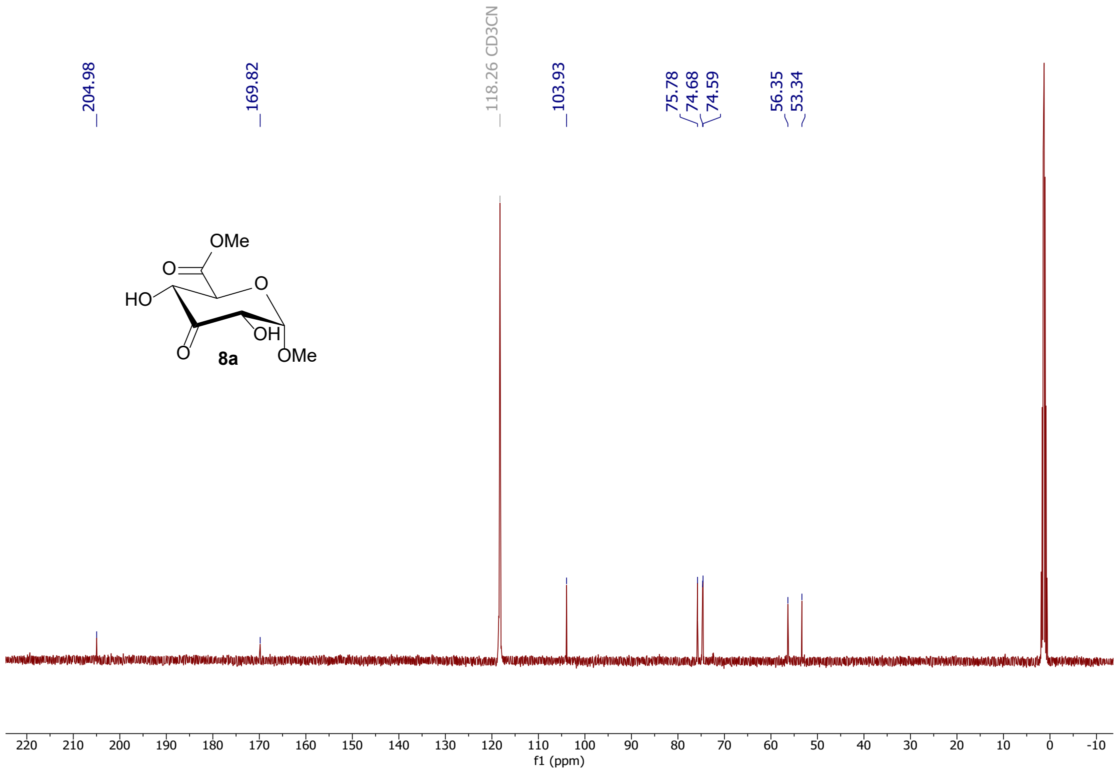
204.98
169.82
118.26 CD3CN
103.93
75.78
74.68
74.59
56.35
53.34
OMe
O
HO
O
OH
O
8a
OMe
220 210 200 190 180 170 160 150 140 130 120 110 100 90 80 70 60 50 40 30 20 10 0 -10
f1 (ppm)

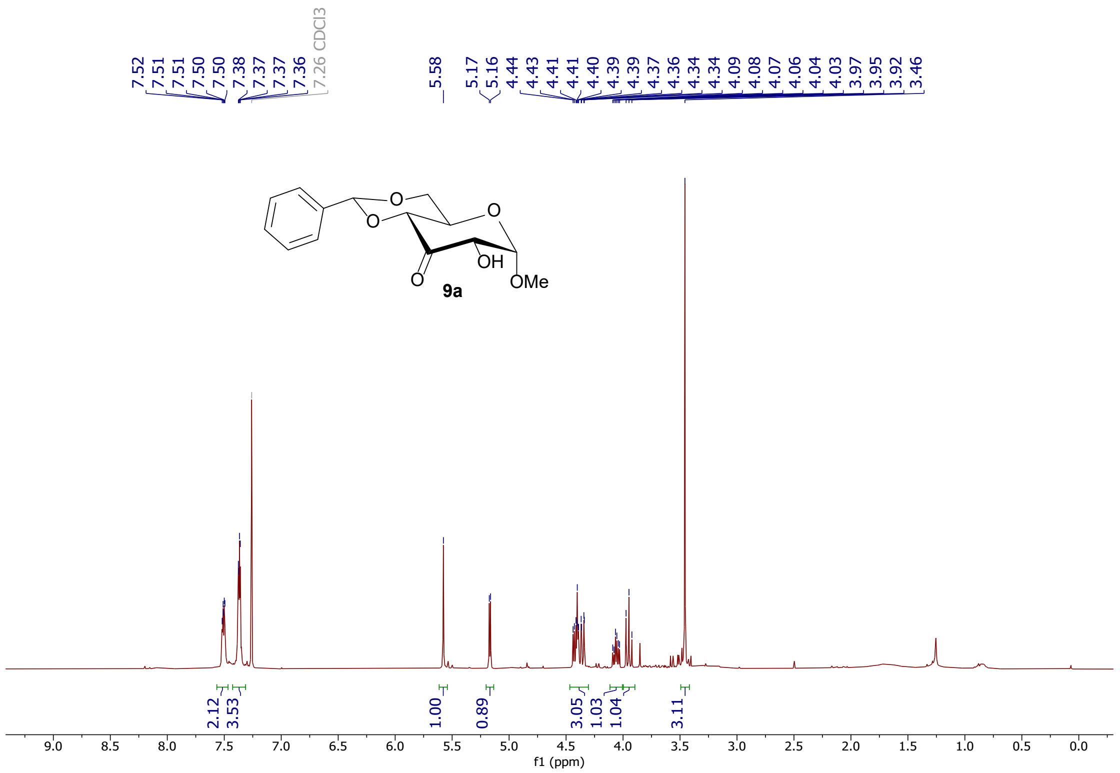
7.52 7.51 7.51 7.50 7.50 7.38 7.37 7.37 7.36 7.26 CDCl3
5.58
5.17 5.16 4.44 4.43 4.41 4.41 4.40 4.39 4.39 4.37 4.36 4.34 4.34 4.09 4.08 4.07 4.06 4.04 4.03 3.97 3.95 3.92 3.46
O
O
O
OH
O
OMe
9a
2.12 3.53 1.00 0.89 3.05 1.03 1.04 3.11
f1 (ppm)

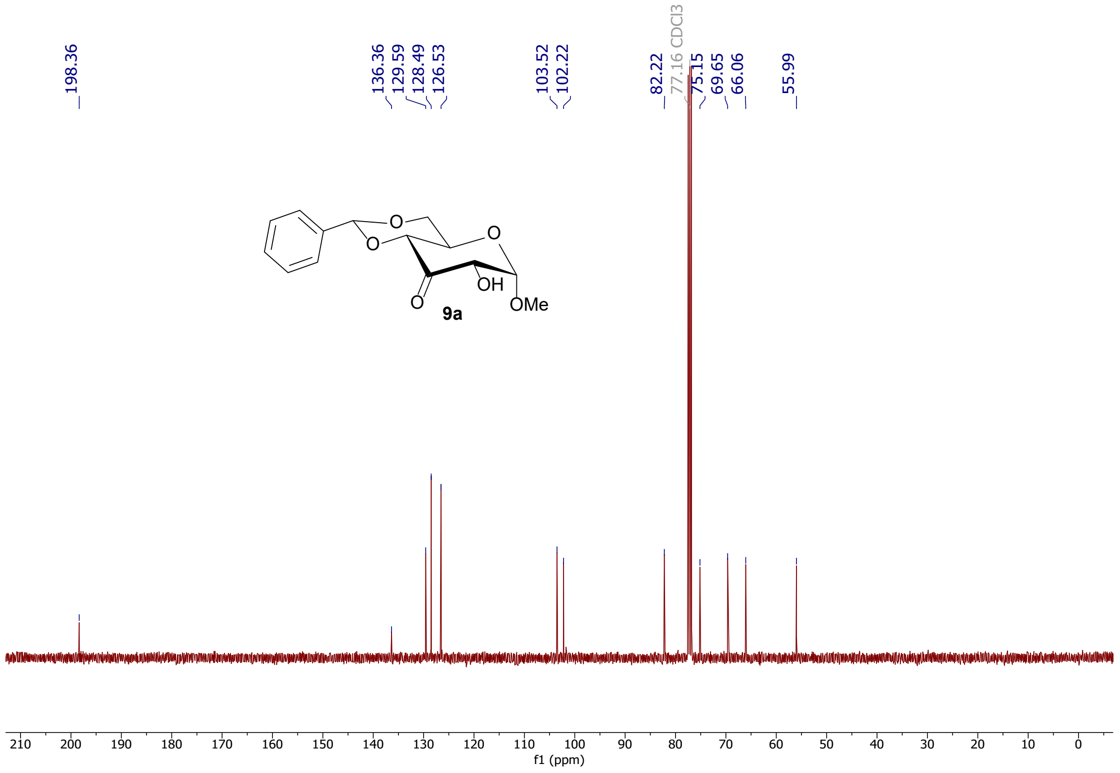
198.36
136.36 129.59 128.49 126.53
103.52 102.22
82.22 77.16 CDCl3 75.15 69.65 66.06
55.99
O
O
O
OH
O
OMe
9a
f1 (ppm)

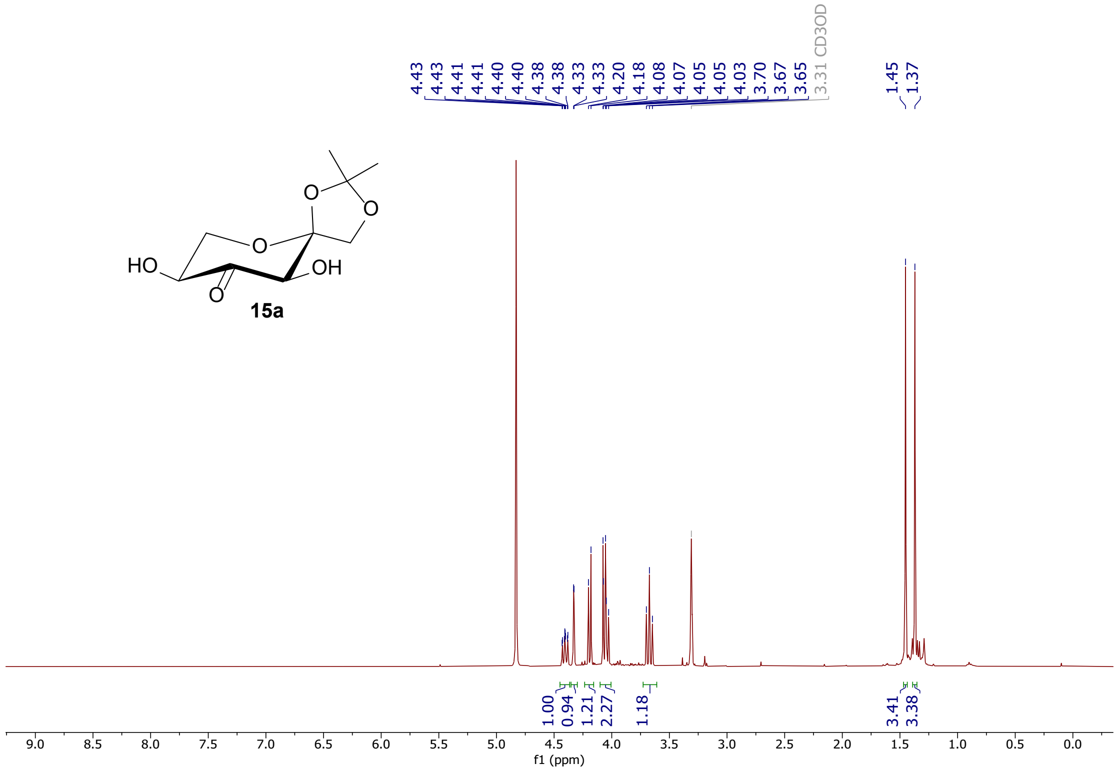

4.43
4.43
4.41
4.41
4.40
4.40
4.38
4.38
4.33
4.33
4.20
4.18
4.08
4.07
4.05
4.05
4.03
3.70
3.67
3.65
3.31 CD3OD
1.45
1.37
HO
O
O
O
OH
O
15a
1.00
0.94
1.21
2.27
1.18
3.41
3.38
9.0
8.5
8.0
7.5
7.0
6.5
6.0
5.5
5.0
4.5
4.0
3.5
3.0
2.5
2.0
1.5
1.0
0.5
0.0
f1 (ppm)

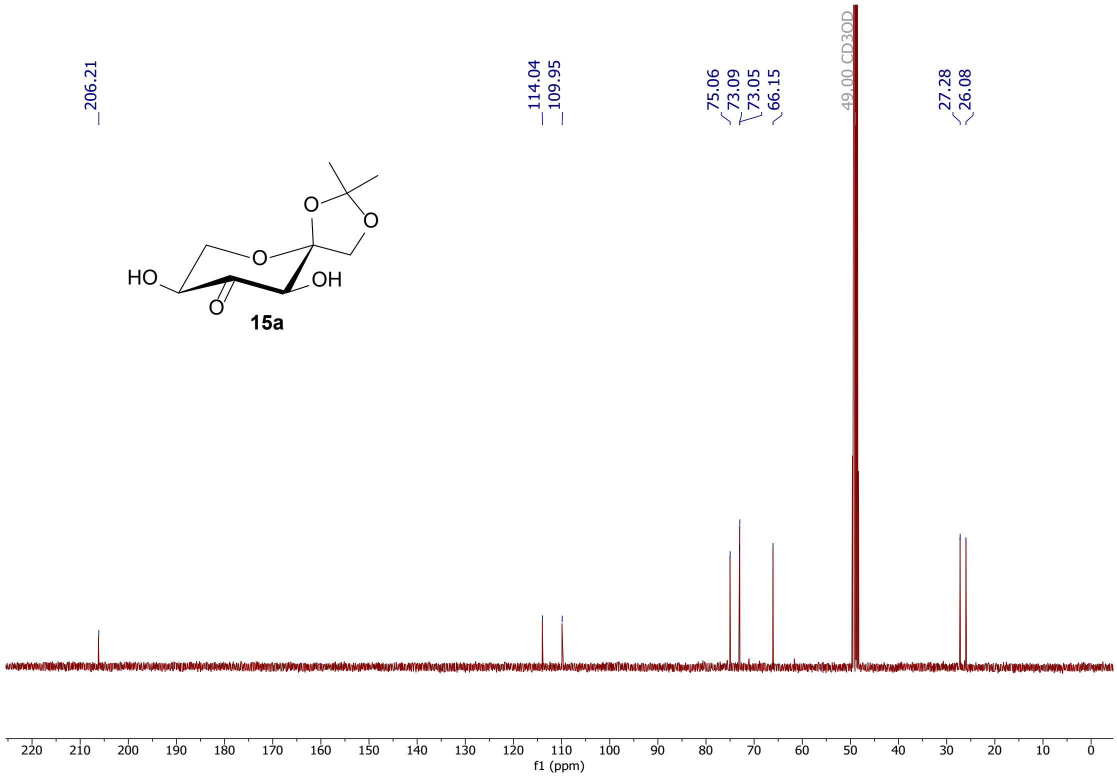

206.21
114.04
109.95
75.06
73.09
73.05
66.15
49.00 CD3OD
27.28
26.08
HO
O
O
O
OH
O
15a
220
210
200
190
180
170
160
150
140
130
120
110
100
90
80
70
60
50
40
30
20
10
0
f1 (ppm)

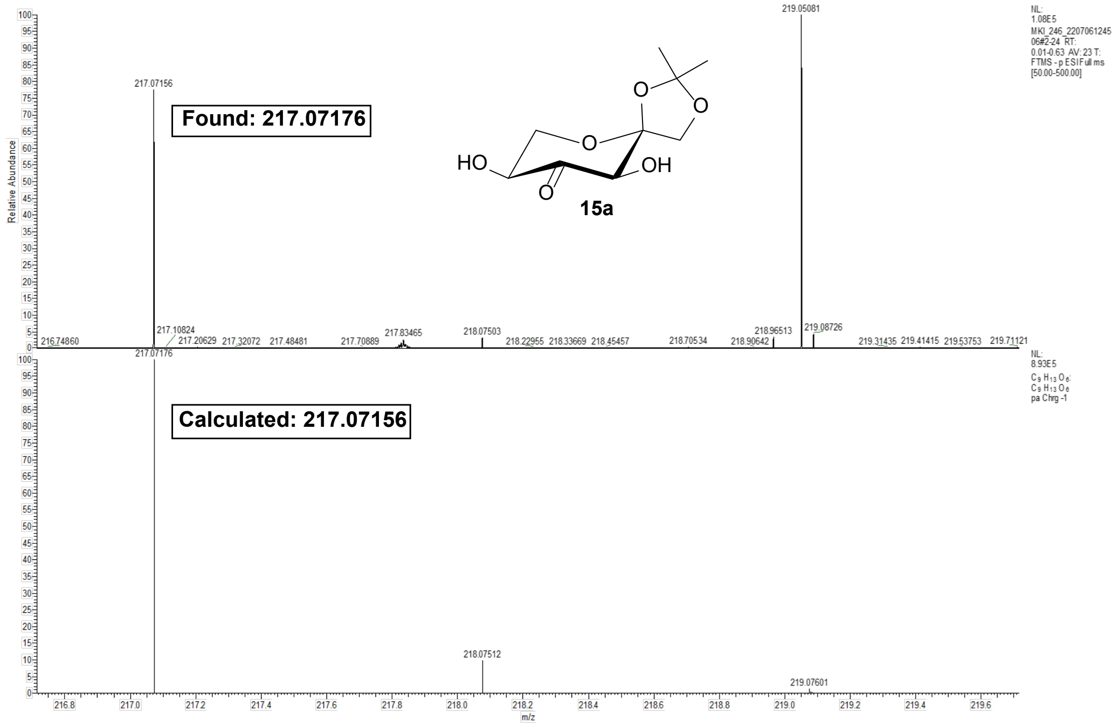
Found: 217.07176
Calculated: 217.07156
15a
HO
OH
Relative Abundance
m/z
NL: 1.08E5
MKI_246_2207061245 06#2-24 RT: 0.01-0.63 AV: 23 T: FTMS - p ESI Full ms [50.00-500.00]
NL: 8.93E5
C9 H13 O6: C9 H13 O6
pa Chrg -1
217.07156
219.05081
217.07176
218.07512
219.07601

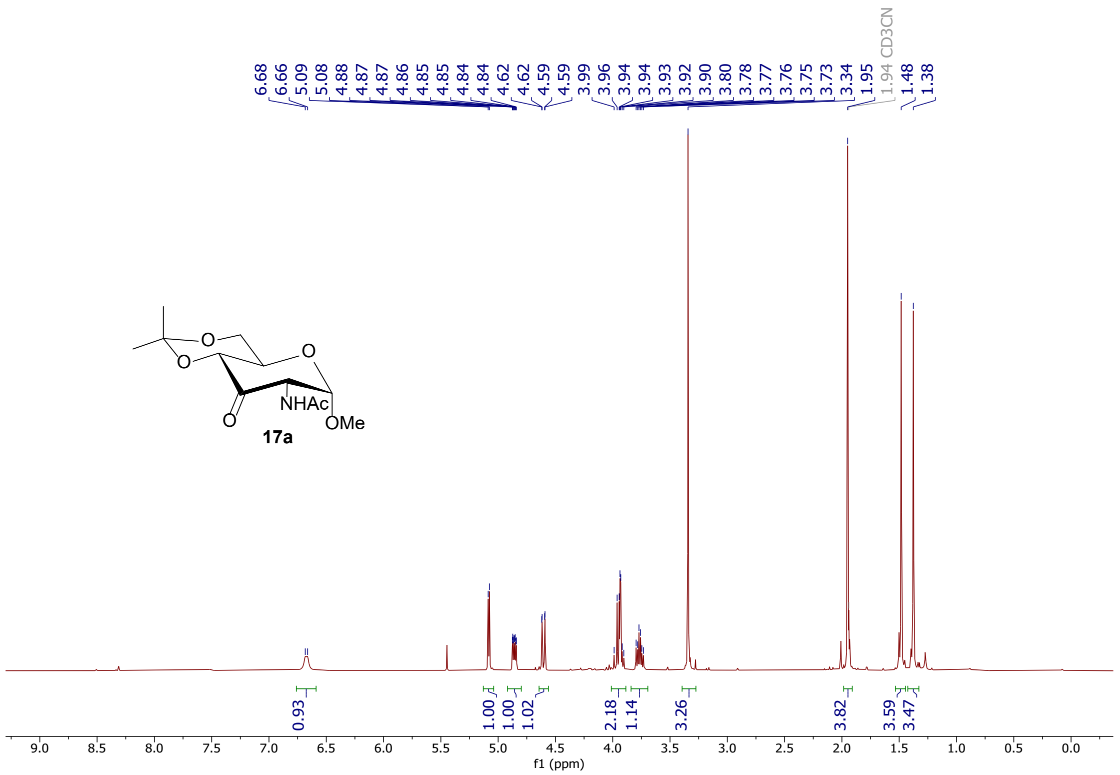
6.68 6.66 5.09 5.08 4.88 4.87 4.87 4.86 4.85 4.85 4.84 4.84 4.62 4.59 4.59 3.99 3.96 3.94 3.94 3.93 3.92 3.90 3.80 3.78 3.77 3.76 3.75 3.73 3.34 1.95 1.94 CD3CN 1.48 1.38
O
O
O
NHAc
O
OMe
17a
0.93 1.00 1.00 1.02 2.18 1.14 3.26 3.82 3.59 3.47
9.0 8.5 8.0 7.5 7.0 6.5 6.0 5.5 5.0 4.5 4.0 3.5 3.0 2.5 2.0 1.5 1.0 0.5 0.0
f1 (ppm)

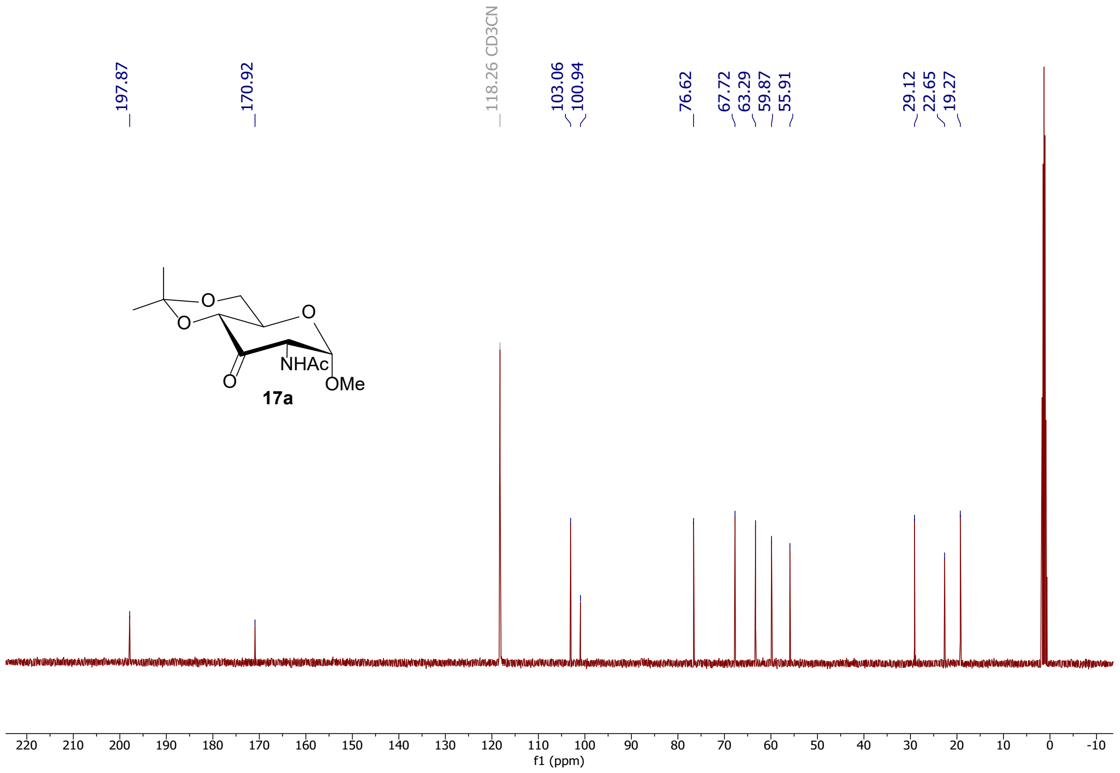
197.87
170.92
118.26 CD3CN
103.06 100.94
76.62
67.72 63.29 59.87 55.91
29.12 22.65 19.27
O
O
O
NHAc
O
OMe
17a
220 210 200 190 180 170 160 150 140 130 120 110 100 90 80 70 60 50 40 30 20 10 0 -10
f1 (ppm)

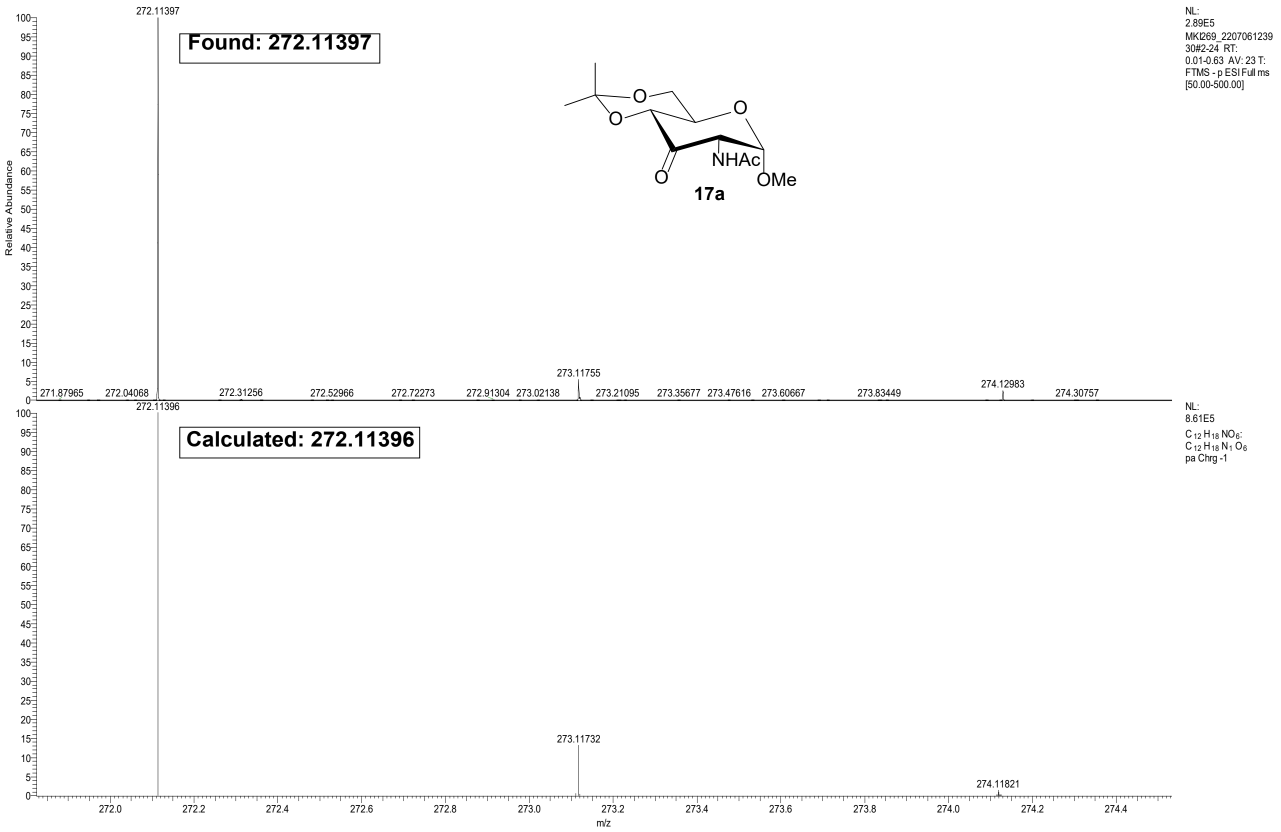
Found: 272.11397
Calculated: 272.11396
NHAc
OMe
17a
NL: 2.89E5
MKI269_2207061239 30#2-24 RT: 0.01-0.63 AV: 23 T: FTMS - p ESI Full ms [50.00-500.00]
NL: 8.61E5
C 12 H 18 NO 6: C 12 H 18 N 1 O 6
pa Chrg -1
Relative Abundance
m/z

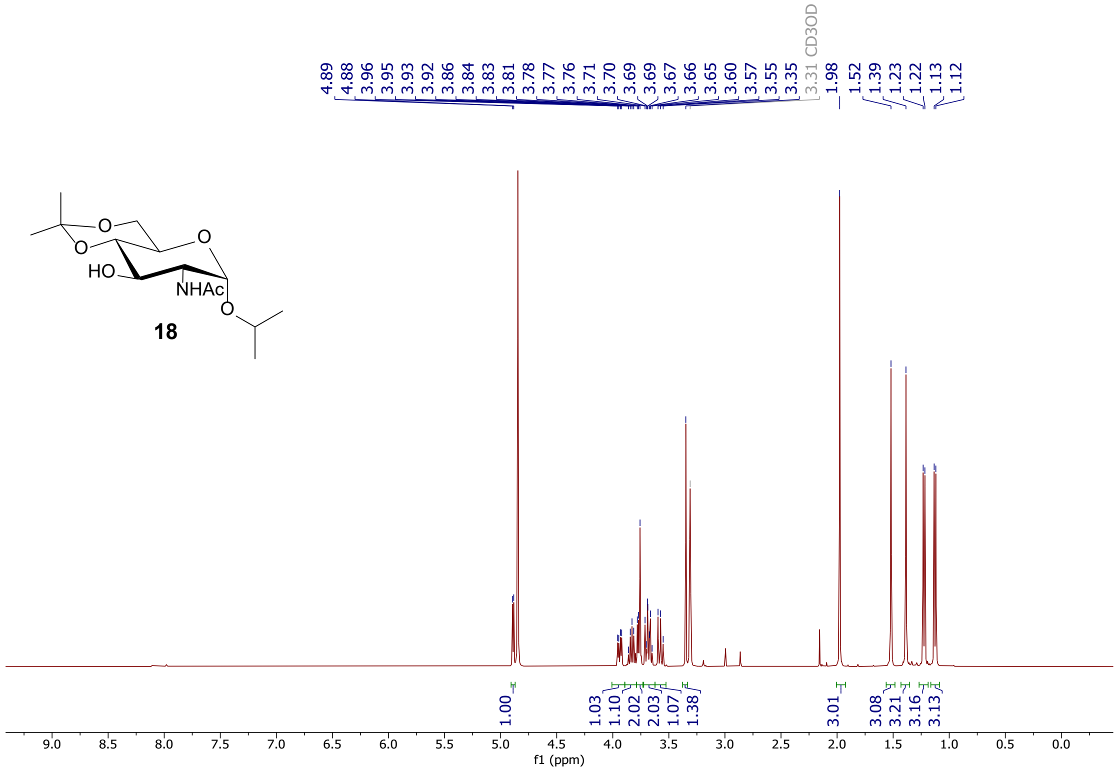
4.89 4.88 3.96 3.95 3.93 3.92 3.86 3.84 3.83 3.81 3.78 3.77 3.76 3.71 3.70 3.69 3.69 3.67 3.66 3.65 3.60 3.57 3.55 3.35
3.31 CD3OD
1.98 1.52 1.39 1.23 1.22 1.13 1.12
O
O
O
HO
NHAc
O
18
1.00 1.03 1.10 2.02 2.03 1.07 1.38 3.01 3.08 3.21 3.16 3.13
9.0 8.5 8.0 7.5 7.0 6.5 6.0 5.5 5.0 4.5 4.0 3.5 3.0 2.5 2.0 1.5 1.0 0.5 0.0
f1 (ppm)

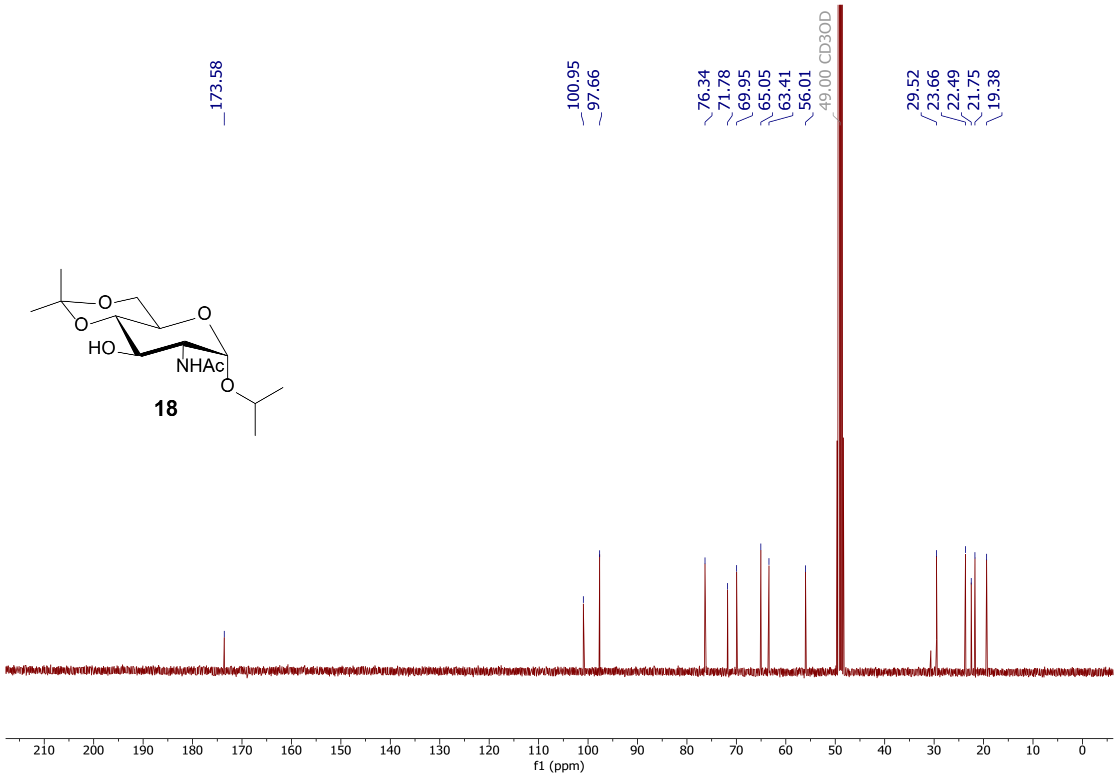
173.58
100.95 97.66
76.34 71.78 69.95 65.05 63.41 56.01
49.00 CD3OD
29.52 23.66 22.49 21.75 19.38
O
O
O
HO
NHAc
O
18
210 200 190 180 170 160 150 140 130 120 110 100 90 80 70 60 50 40 30 20 10 0
f1 (ppm)

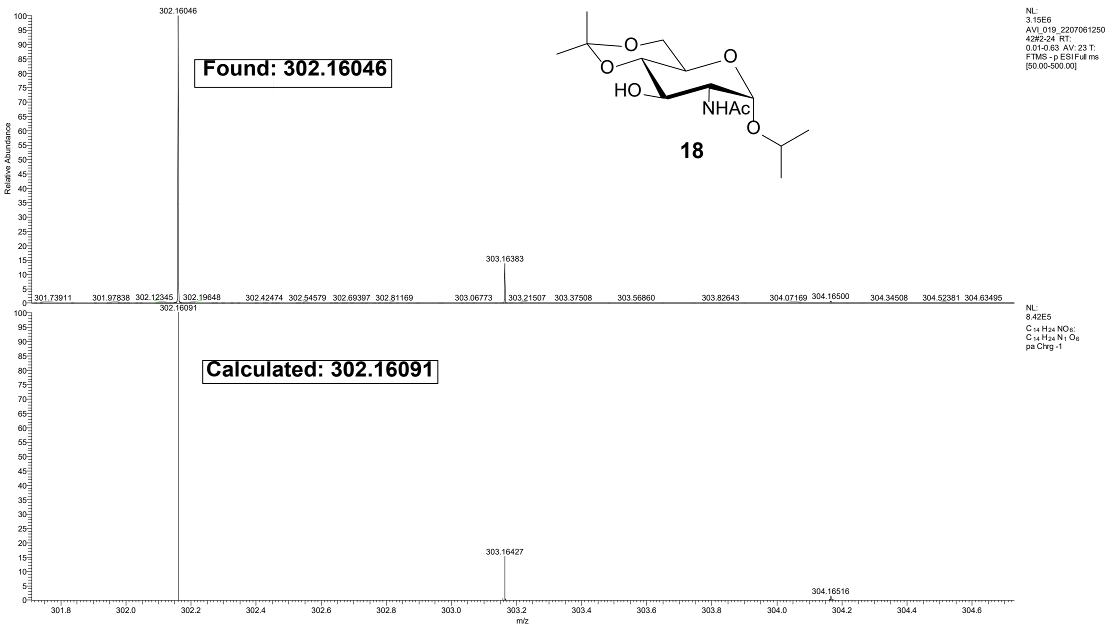
Found: 302.16046
Calculated: 302.16091
HO
NHAc
18
Relative Abundance
m/z
NL: 3.15E6
AVI_019_2207061250 42#2-24 RT: 0.01-0.63 AV: 23 T: FTMS - p ESI Full ms [50.00-500.00]
NL: 8.42E5
C 14 H24 NO6: C 14 H24 N1 O6 pa Chrg -1

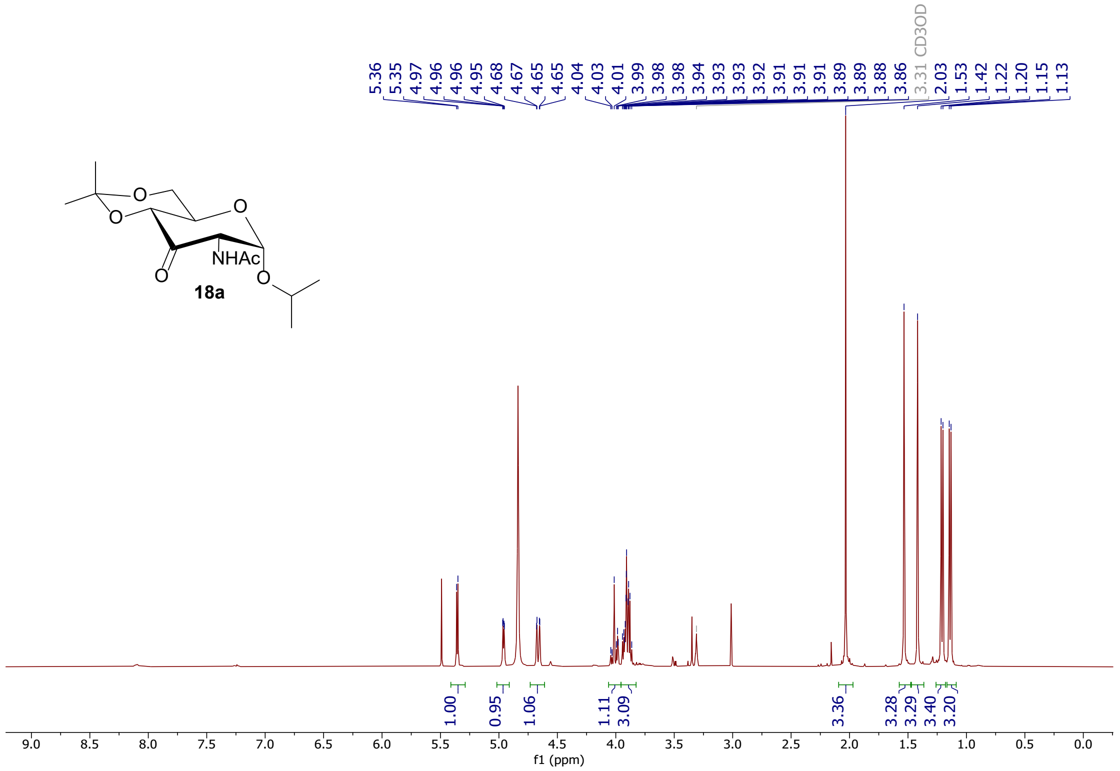
5.36
5.35
4.97
4.96
4.96
4.95
4.68
4.67
4.65
4.65
4.04
4.03
4.01
3.99
3.98
3.98
3.94
3.93
3.93
3.92
3.91
3.91
3.91
3.89
3.89
3.88
3.86
3.31 CD3OD
2.03
1.53
1.42
1.22
1.20
1.15
1.13
NHAc
O
18a
1.00
0.95
1.06
1.11
3.09
3.36
3.28
3.29
3.40
3.20
f1 (ppm)

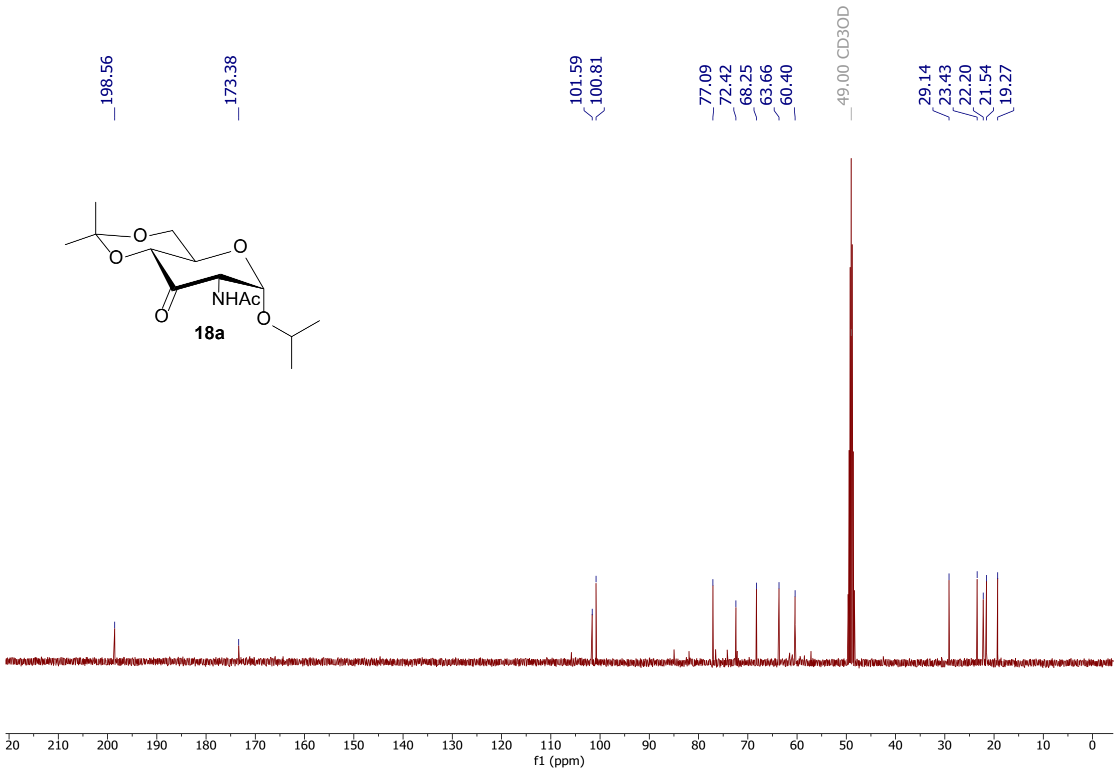
198.56
173.38
101.59
100.81
77.09
72.42
68.25
63.66
60.40
49.00 CD3OD
29.14
23.43
22.20
21.54
19.27
NHAc
O
18a
f1 (ppm)

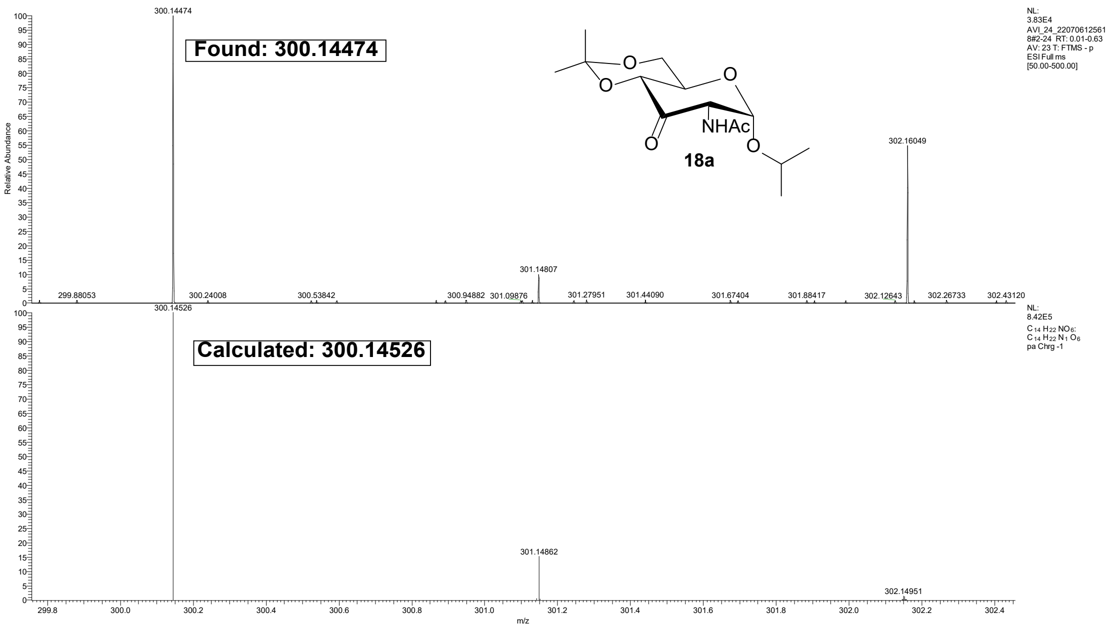
Found: 300.14474
Calculated: 300.14526
18a
NHAc
NL: 3.83E4
AVI_24_22070612561 8#2-24 RT: 0.01-0.63 AV: 23 T: FTMS - p ESI Full ms [50.00-500.00]
NL: 8.42E5
C 14 H22 NO6:
C 14 H22 N1 O6
pa Chrg -1
Relative Abundance
m/z

References

1. Kasun, Z. A.; Geary, L. M.; Krische, M. J., Ring expansion of cyclic 1,2-diols to form medium sized rings via ruthenium catalyzed transfer hydrogenative [4+2] cycloaddition. *Chem. Commun.* **2014**, *50* (56), 7545–7547.
2. Corey, E. J.; Venkateswarlu, A., Protection of hydroxyl groups as tert-butyldimethylsilyl derivatives. *J. Am. Chem. Soc.* **1972**, *94* (17), 6190–6191.
3. Wan, I. C. S.; Witte, M. D.; Minnaard, A. J., From d- to l-Monosaccharide Derivatives via Photodecarboxylation–Alkylation. *Org. Lett.* **2019**, *21* (18), 7669–7673.
4. Lee, A. A.; Chen, Y.-C. S.; Ekalestari, E.; Ho, S.-Y.; Hsu, N.-S.; Kuo, T.-F.; Wang, T.-S. A., Facile and Versatile Chemoenzymatic Synthesis of Enterobactin Analogues and Applications in Bacterial Detection. *Angew. Chem. Int. Ed.* **2016**, *55* (40), 12338–12342.
5. Eisink, N. N. H. M.; Witte, M. D.; Minnaard, A. J., Regioselective Carbohydrate Oxidations: A Nuclear Magnetic Resonance (NMR) Study on Selectivity, Rate, and Side-Product Formation. *ACS Catal.* **2017**, *7* (2), 1438–1445.
6. Lochyński, S.; Frąckowiak, B.; Olejniczak, T.; Ciunik, Z.; Wawrzeńczyk, C., Lactones. Part 15: Synthesis of chiral spirolactones with a carane system—insect feeding deterrents. *Tetrahedron: Asymmetry* **2002**, *13* (16), 1761–1767.
7. Corkran, H. M.; Munneke, S.; Dangerfield, E. M.; Stocker, B. L.; Timmer, M. S. M., Applications and Limitations of the I2-Mediated Carbamate Annulation for the Synthesis of Piperidines: Five- versus Six-Membered Ring Formation. *J. Org. Chem.* **2013**, *78* (19), 9791–9802.
8. Gizaw, Y.; BeMiller, J. N., Application of a phase transfer reaction to the synthesis of l-fructose. *Carbohydrate Research* **1995**, *266* (1), 81–85.
9. Zhang, J.; Eisink, N. N. H. M.; Witte, M. D.; Minnaard, A. J., Regioselective Manipulation of GlcNAc Provides Allosamine, Lividosamine, and Related Compounds. *J. Org. Chem.* **2019**, *84* (2), 516–525.
10. Kovac, P.; Edgar, K. J., Synthesis of ligands related to the O-specific antigen of type 1 Shigella dysenteriae. 3. Glycosylation of 4,6-O-substituted derivatives of methyl 2-acetamido-2-deoxy-α-D-glucopyranoside with glycosyl donors derived from mono- and oligosaccharides. *J. Org. Chem.* **1992**, *57* (8), 2455–2467.
11. Ishida, H.; Ogawa, Y.; Imai, Y.; Kiso, M.; Hasegawa, A.; Sakurai, T.; Azuma, I., Chemical combination of 6-deoxy-6-mycoloylamino-α,α-trehalose and N-acetyl-6-O-(aminoacyl)muramoyl dipeptide. *Carbohydrate Research* **1989**, *194*, 199–208.
12. Marinus, N.; Tahiri, N.; Duca, M.; Mouthaan, L. M. C. M.; Bianca, S.; van den Noort, M.; Poolman, B.; Witte, M. D.; Minnaard, A. J., Stereoselective Protection-Free Modification of 3-Keto-saccharides. *Org. Lett.* **2020**, *22* (14), 5622–5626.
13. Gorelik, D. J.; Dimakos, V.; Adrianov, T.; Taylor, M. S., Photocatalytic, site-selective oxidations of carbohydrates. *Chem. Commun.* **2021**, *57* (91), 12135–12138.
14. Defaye, J.; Gadelle, A., Oxydation sélective de diols vicinaux secondaires par le réactif diméthylsulfoxyde—anhydride acétique. *Carbohydrate Research* **1974**, *35* (1), 264–269.
